# Advanced Biocompatible and Biodegradable Polymers: A Review of Functionalization, Smart Systems, and Sustainable Applications

**DOI:** 10.3390/polym17212901

**Published:** 2025-10-30

**Authors:** Latifat Abdulsalam, Sadam Abubakar, Ikfa Permatasari, Anas Abdulwahab Lawal, Shihab Uddin, Saleem Ullah, Irshad Ahmad

**Affiliations:** 1 Department of Bioengineering, King Fahd University of Petroleum and Minerals (KFUPM), Dhahran 31261, Saudi Arabia; g202210360@kfupm.edu.sa (L.A.); g202315670@kfupm.edu.sa (S.A.); g202215280@kfupm.edu.sa (I.P.); g202404880@kfupm.edu.sa (A.A.L.); shihab.uddin@kfupm.edu.sa (S.U.); 2 VTT Technical Research Centre of Finland Ltd., Tekniikantie 21, 02150 Espoo, Finland; saleem.ullah@vtt.fi; 3 Interdisciplinary Research Center for Membranes and Water Security, King Fahd University of Petroleum and Minerals (KFUPM), Dhahran 31261, Saudi Arabia

**Keywords:** biocompatible polymers, biodegradable polymers, drug delivery, tissue engineering, sustainable materials, smart polymers

## Abstract

The growing dependence on plastics is driving a sharp increase in environmental pollution, posing serious risks to human health. This issue necessitates immediate attention and proactive measures to mitigate its impact on both individuals and the broader ecosystem. From this viewpoint, biocompatible and biodegradable polymers, both synthetic and natural, have emerged as vital materials for applications in biomedicine, packaging, and environmental sustainability. The main advantages of biodegradable polymer materials lie in conserving fossil fuel resources, utilizing inedible biomass, and enabling environmentally friendly production processes. In this context, this review thoroughly discusses the categorization of biocompatible and biodegradable polymers into natural and synthetic types, detailing their structural characteristics, mechanisms of biodegradation, and compatibility matrices appropriate for biomedical, environmental, and industrial uses. It also addresses recent advancements in polymer synthesis technology, highlighting significant progress in polymer functionalization, responsiveness to stimuli, and environmentally friendly biobased synthesis methods. Additionally, it identifies challenges such as mechanical constraints, control over degradation, and expense, while also discussing future opportunities in the field of polymer science.

## 1. Introduction

Since the 1950s, the study and commercial application of polymers have grown significantly, becoming essential in almost every facet of everyday life. By the late 1980s, worldwide polymer production had exceeded that of steel, and it now totals approximately 413.8 million tons each year [1]. The biggest portion is allocated for packaging (exceeding 30%), while the building and construction sector follows closely with over 20%. The automotive, electrical, and electronic sectors each represent 7% to 10%. The medical industry utilizes less than 2% by weight, which is split between packaging materials (like prefilled syringes and blood bags) and medical devices [1,2].

The extensive application of polymers can be credited to their distinctive characteristics, such as ease of production, low weight, flexibility in mechanics, chemical resilience (especially at low temperatures), and affordability. However, these benefits have also led to considerable environmental issues. Globally, only around 9% of plastic waste is recycled, with the majority either going to landfills or being incinerated, while plastic’s total greenhouse gas emissions from production to disposal were estimated to be 1.8 gigatons of CO_2_ equivalent in 2019, making up roughly 3.4% of the worldwide figure. Incineration of waste is one of the most emission-heavy methods of disposal, and unmanaged leakage continues to negatively impact ecosystems. These challenges have prompted a move toward circular strategies in the realms of design, manufacturing, usage, and final disposal [3].

In response to these issues, both research and industry have sped up the creation of alternatives that minimize hazards, energy use, and waste production. One key approach focuses on the advancement of bio-based and biodegradable polymers intended to perform efficiently for a specified time before decomposing into simpler substances that can be managed and disposed of more easily through regulated methods [4]. It is estimated that in 2022, the production of bioplastic reached 2.22 million tons, with approximately 1.1 million tons (about 51%) categorized as biodegradable plastics, including polylactic acid (PLA), polyhydroxyalkanoates (PHAs), starch blends, polybutylene adipate terephthalate (PBAT), cellulose films, and polybutylene succinate (PBS). Additionally, nearly 1.1 million tons (roughly 49%) consisted of non-biodegradable polymers sourced from biological materials, primarily featuring bio-based polyethylene (PE), polyethylene terephthalate (PET), polyamides (PA), and polypropylene (PP), among others [5].

Biodegradable polymers are widely applied in biomedical fields, including tissue engineering, regenerative medicine, urology, controlled drug delivery, cardiac surgery, dentistry, and orthopedics, among others [6]. Literature reviews have highlighted the increasing utilization of these materials in controlled drug delivery systems, temporary implants, tissue scaffolds, and resorbable devices, where the polymer chemistry, processing methods, and microstructure are tailored to meet specific biocompatibility and degradation requirements [7,8,9].

This article explores the key properties of polymers, including their degradation mechanisms, biocompatibility, and mechanical and thermal characteristics, and categorizes polymers based on their origin as either natural or synthetic. The manuscript highlights recent technological advancements aimed at improving polymer interactions with biological systems, enhancing functional performance, and promoting sustainable, bio-based synthesis methods. Additionally, it examines the critical roles of polymers in medical, environmental, and industrial applications. Finally, the article addresses current challenges, limitations, and potential directions for future research in the field.

## 2. Key Properties and Classification of Biopolymers

### 2.1. Key Physicochemical and Biological Properties

The successful application of biopolymers in environmental and biomedical settings relies heavily on a thorough understanding of their physicochemical and biological properties. These properties influenced how they interact and degrade under different conditions. The key properties that are pivotal for the application of biopolymers include degradation mechanism, biocompatibility, and mechanical and thermal properties.

#### 2.1.1. Degradation Mechanisms

The pathways of polymer degradation are crucial factors that shape their performance in environmental and biomedical applications. Biopolymers mainly degrade through hydrolytic and enzymatic degradation pathways, although other mechanisms, particularly photodegradation, are also possible.

In hydrolytic degradation, water molecules often cleave the bonds that join the monomeric units in polymers. A typical example is the cleavage of the ester bonds in PLA by a water molecule [10].

Various factors, including humidity, temperature, and catalyst availability, greatly influence the hydrolytic degradation pathways of biopolymers. For example, the hydrolysis rate of PLA can increase by 30–50% when the temperature is raised by 50 °C under humidity above 90%, compared to the hydrolysis rate at normal environmental conditions. Similarly, the presence of 0.5% by weight of SnCl_2_ has been shown to accelerate PLA hydrolysis by approximately 40% relative to pure PLA, under the same environmental temperature and humidity conditions [11].

Enzymatic degradation occurs due to the action of enzymes that cleave the bonds joining the monomeric units in the polymer backbone. For example, the enzymatic cleavage of α-1,4-glycosidic linkages in starch-based polymers. Specifically, enzymes such as β-glucosidase and α-amylase act on the α-1,4-glycosidic linkages in starch-based polymers, whereas enzymes such as lipases, proteases, and esterases act on the ester bonds in PLA [10]. Similarly to hydrolytic degradation pathways, the enzymatic degradation of polymer can also be strongly influenced by factors such as temperature and humidity. A typical example shows how the rate of enzymatic degradation is accelerated by raising the temperature from 30 °C to 50 °C and maintaining humidity above 80% [12].

Overall, both hydrolytic and enzymatic degradation mechanisms play significant roles in determining the lifespan and stability of biopolymers in environmental and biomedical applications.

#### 2.1.2. Biocompatibility Metrics

The thorough assessment of the biocompatibility of biodegradable and biocompatible polymers is essential. This is particularly important when these biopolymers are intended to be used in human beings. Biomaterials are typically evaluated for toxicity, allergic potential, and immunogenicity to prevent adverse events and ensure patient safety. Additionally, several regulatory bodies require rigorous biocompatibility testing before such materials are commercialized for consumer or clinical applications. Biocompatibility evaluation involves multiple aspects, including the careful selection of test types, choice of relevant cell lines or tissues, and performance assays tailored to the intended function of the biomaterials [13].

While most biopolymers discussed are generally regarded as biocompatible, a long-term biocompatibility assessment remains crucial. For example, PEG has traditionally been considered a non-immunogenic and biocompatible biomaterial. However, Chen et al. (2025) [14] reported the presence of anti-PEG antibodies, either pre-existing or induced by PEGylated vaccines and medicine, that may compromise the safety and efficacy of nanomedicines. These antibodies can alter the biodistribution of nanocarriers, stimulate undesirable inflammatory and hypersensitivity responses, and destabilize lipid formulations [14]. Similarly, PLA is also regarded as a biomaterial with good biocompatibility. However, when applied in vivo, it can provoke an inflammatory reaction and adverse tissue responses. Liu et al. (2024) reported that PLA-based microspheres modified with short-chain PEG exhibited enhanced histocompatibility [15].

#### 2.1.3. Mechanical and Thermal Characteristics

The mechanical and thermal properties of biopolymers are crucial factors to consider for their suitability, especially for biomedical applications. From a mechanical characteristics perspective, the inherent biodegradability and biocompatibility properties of polymers render them suitable for use in bone tissue engineering and wound healing. For example, natural polymers such as collagen, alginate, and chitosan offer unique biocompatibility and biodegradability properties. They also promote cell proliferation and adhesion because of their structural similarities to the extracellular matrix (ECM). However, their practical applications, especially in load-bearing applications such as scaffolds for bone healing, are limited due to their low mechanical strength. As such, natural biopolymers are often blended with synthetic polymers or reinforced with inorganic substances such as calcium phosphates to improve their mechanical properties [16,17].

Conversely, synthetic polymers, particularly PLA and PCL, often exhibit impressive tunability and favorable mechanical properties. These properties allow synthetic polymers to be more suitable for long-term load-bearing applications compared to natural biopolymers. However, their lack of natural bioactivity often limits broader biomedical applications. As such, to improve their bioactivity for cell attachment and osteoconductivity, synthetic biopolymers are usually modified by combining them with bioactive materials [17].

Other important properties for biomedical and environmental applications of biopolymers beyond mechanical properties are the thermal properties. For example, the suitability of a biopolymer-based packaging film in food storage and preservation largely depends on its thermal stability. This ensures that the polymeric material maintains both its functional and structural integrity within a range of temperatures. As such, thermal stability prevents the premature degradation of polymeric packaging films, ensuring adequate protection of food products during processing, transportation, and storage.

To assess the thermal properties, analytical techniques such as differential scanning calorimetry (DSC), thermogravimetric analysis (TGA), and thermomechanical analysis are often invaluable. These techniques provide detailed insight into the relationship between polymeric packaging films and their thermal behavior and material structure, including crystallinity, chemical arrangements, morphology, and the role of nano-reinforcements in enhancing the thermal stability of polymeric films [18]. A notable study introduces PCL into PLA/PCL blends at varying ratios, which has been shown to influence the thermal properties, degradation rate, and flexibility of a 3D printed scaffold, demonstrating the importance and potential of this blend in tailoring material properties for specific tissue engineering applications [19].

Following this comprehensive overview of the fundamental physicochemical and biological properties that influence the behaviors of biopolymers, we next discuss the classification of natural and synthetic polymers. Throughout, we emphasize comparative performance, limitations, and specific design considerations, with concise summaries in Table 1 (natural polymers) and Table 2 (synthetic polymers).

### 2.2. Natural and Synthetic Polymers

Biodegradable polymers refer to polymeric materials that can be decomposed or disintegrated primarily through the enzymatic action of microorganisms into useful environmental products, such as water, methane, biomass, and carbon dioxide. On the other hand, other polymeric materials are considered biocompatible, meaning they do not elicit toxic, inflammatory, or immunogenic responses, particularly when used as biomaterials in humans. Both biodegradable and biocompatible polymers are used in diverse sectors, including biomedical devices and drug delivery, sustainable packaging, and agriculture [13,20,21,22].

For clarity, in this review, polymers are classified as natural or synthetic based on their source, irrespective of whether they are biodegradable, biocompatible, or both.

**Figure 1:**
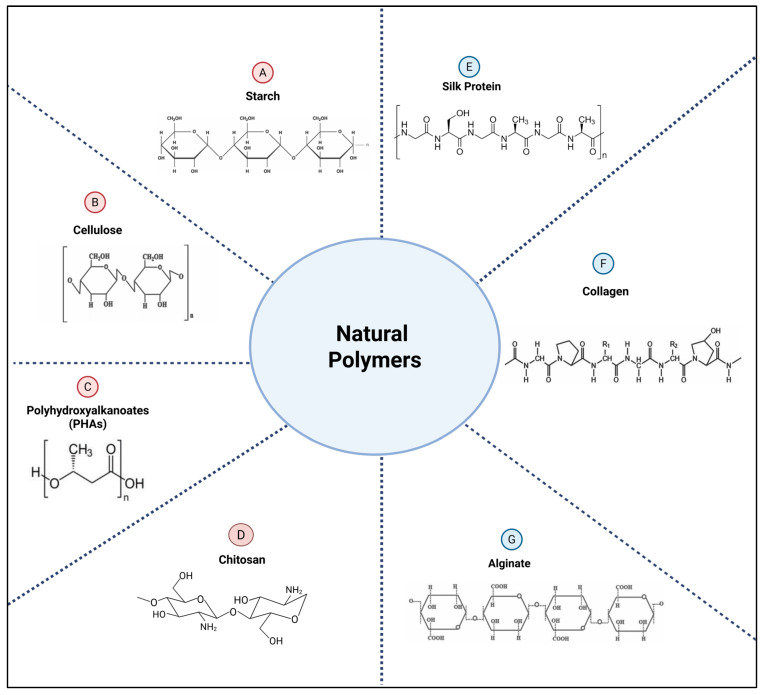
Representative structures of natural polymers. Illustration showing the chemical structures of common natural polymers, including starch, cellulose, PHAs, chitosan, silk protein, collagen, and alginate. Created with Biorender.com.

**Figure 2:**
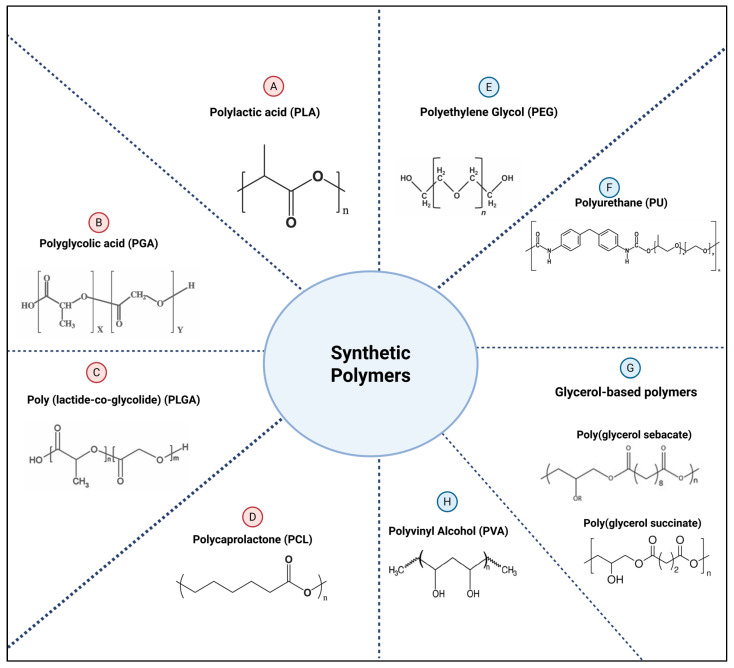
Representative structures of synthetic polymers. Illustration showing the chemical structures of common synthetic polymers, including PGA, PLGA, PLA, PEG, PU, glycerol-based polymers, PVA and PCL. Created with Biorender.com.

Natural polymers, as the name suggests, are derived from natural sources (Figure 1), while synthetic polymers are artificially synthesized to meet a particular need (Figure 2). This classification provides the framework for comparing the performance, limitations, and design considerations of each polymer across the two subclasses.

#### 2.2.1. Natural Polymers

- Starch

Starch is one of the most abundant and inexpensive biodegradable polymers. It is a low-cost hydrophilic polysaccharide produced in the form of granules by several crops. The main crops from which starch is extracted include corn, rice, potatoes, and wheat. Chemically, starch is composed of amylose (linear) and amylopectin (branching) poly-(1,4)-α-glucose, and its physicochemical properties are strongly influenced by the amylose-to-amylopectin ratio [23]. Starch is poorly soluble in water at ambient temperature. However, the solubility of starch largely depends on the relative proportions of amylose to amylopectin [24,25,26].

However, some drawbacks of starch for broad applications include water sensitivity, brittleness, poor mechanical strength, and low impact resistance. Consequently, it is a common practice to chemically or physically modify starch to enhance its properties for a specific application. Due to their renewability, excellent oxygen barrier properties, and high biodegradability, starch-based biopolymers are utilized in various applications, including biomedical, packaging, agricultural, and adhesives [27]. These properties make starch-based films attractive for niche packaging applications, such as pouches, single-use wraps, and coating layers for vegetables, fruits, or snacks [28].

Despite the promising potential of starch-based films, the brittleness and high moisture absorption ability of starch impair its film-forming capacity. These limitations are often addressed by incorporating plasticizers into the starch matrix to enhance the water resistance and flexibility. However, adding plasticizers such as fructose and glycerol to starch-based films typically enhances ductility and water stability at the expense of mechanical strength. For instance, with the addition of 35% fructose, the tensile strength of wheat starch films decreases from 38.7 MPa to 7.6 MPa [29,30].

Recent reviews highlight the application of starch-based hydrogels (SBHs) in cutting-edge fields, including drug delivery, flexible sensors, wound dressings, tissue engineering, food packaging, soil protection, and wastewater treatment [31]. As a biomedical application example, a pH-responsive starch/PVA/g-C_3_N_4_ nanocarrier hydrogel has been shown to enhance doxorubicin delivery to cancer tumors or in vitro breast cancer cells, indicating the potential of SBHs for targeted therapy [32]. However, the weak mechanical properties of pure starch-based hydrogels have prompted the need for innovative strategies to enhance them. This is commonly achieved through the addition of nanofibers, inorganic fillers, or various crosslinking methods [33].

**Table 1:** Comparison of key properties of natural polymers.

Polymer	Mechanical Strength	Degradation Rate	Biocompatibility and Biodegradability	Key Limitations	References
Starch	Poor mechanical strength; reinforced with fiber matrix or by chemical or physical modification	Fast; temperature dependent	Biocompatible and biodegradable	Moisture absorbance and mechanical failure	[10,27]
Cellulose	Mechanical property weakened by moisture absorption; improved by chemical modification	Fast; temperature and environmental factors dependent	Biocompatible and biodegradable	Moisture absorbance and deterioration of mechanical properties	[10,27]
Polyhydroxyalkanoates (PHAs)	Weak mechanical property. Brittle.	Relatively fast, depending on HV content	Biocompatible and biodegradable	Commercially available PHAs are still brittle	[27,34,35]
Chitosan	Moderate mechanical properties, 10–60 MPa	Tunable degradation rate, Days-Months	Biocompatible and biodegradable	Poor water resistance, non-thermoplastic	[7,36,37,38]
Silk protein	0.74–1.65 GPa tensile strength (native silk fibers)	Weeks–months; tunable	Biocompatible	Brittle when dry, recombinant yield is low	[39,40,41]
Collagen	50–150 MPa tensile strength, 0.3–1.2 GPa Young’s modulus	Weeks–months (native); days–weeks (marine)	fully biodegradable and highly biocompatible	May be immunogenic and pathogens contaminated	[42,43,44,45,46,47]
Alginate	Poor mechanical properties; may be improved by crosslinking with multivalent cations.	Fast at high temperatures	Biocompatible and biodegradable	Poor stability and mechanical properties. Difficulty in customization.	[48]

Graft engineering can also influence the properties of starch. For instance, a starch-based hydrogel produced by grafting polylactic acid and acrylamide onto starch exhibits antibacterial properties against both Gram-negative and Gram-positive bacteria. In addition, the synthesized hydrogel not only exhibits improved thermal and mechanical properties but is also biodegradable and non-toxic, making it an excellent material for wound dressings [49]. Despite the reported improvement, the long-term stability of grafted SBHs remains unclear and requires further research [33].

Apart from biomedicine, starch and starch-based materials are increasingly used as adsorbents for removing heavy metals and emerging pollutants, including pesticides and antibiotics, in addition to their roles in drug delivery and imaging. However, the promising removal of several pollutants by starch-based absorbents does not signify high selectivity for a specific pollutant. Therefore, the development of pollutant-specific starch-based absorbents remains an area of future research [50].

- Cellulose

Cellulose is a common organic polymer found in the cell walls of organisms from distinct biological groups, including algae, green plants, and bacteria. Chemically, cellulose is composed mainly of glucose monomers linked by β-1,4-glycosidic bonds, in contrast to the α-glycosidic bonds found in starch, a related biopolymer. It can be sourced from natural biological materials, particularly wood, cotton, hemp, and other fibrous plants. Both cellulose and its derivatives are considered eco-friendly materials due to their compatibility with various materials, regenerative properties, and ability to degrade. In addition, cellulose exhibits excellent rigidity, tensile strength, and mechanical properties [27,51].

The favorable mechanical properties of cellulose are associated with its ability to provide structural support, enabling plants to resist mechanical stress [52]. The tensile strength of cellulose-based films ranges from 30 to 50 MPa, while that of starch ranges only from 15 to 40 MPa [53].

However, the mechanical properties of cellulose may be compromised by moisture due to its hydrophilic nature. A common cellulose modification strategy involves the introduction of a specific reagent that reacts with its hydroxyl groups to reduce its hydrophilicity and enhance its mechanical and solubility properties [54].

Both cellulose and its derivatives have been widely applied in various sectors, including pharmaceutical, textile, wood, and fiber industries. A recent study highlights the potential of cellulose as a drug carrier, enhancing both the release of drugs and inhibiting the ability of several bacterial species to form biofilms [51,55]. Despite their promising potential, cellulose-based materials face challenges, including long-term durability, variability in properties, high production costs, and the need to tailor properties for specific applications [56].

- Polyhydroxyalkanoates (PHAs)

Polyhydroxyalkanoates (PHAs) belong to the family of aliphatic biopolyesters that accumulate inside microbial cells as intracellular granules (carbonosomes). When carbon-rich, low-value, or waste substrates are available and other nutrients are limiting (typically nitrogen), some microorganisms can synthesize and store PHAs that are equivalent to 30% to 80% of their cellular dry mass [23,57].

PHAs are thermoplastic, generally biocompatible, and biodegradable biopolymers derived from several renewable feedstocks [58].

PHAs are increasingly considered an innovative alternative to non-degradable plastics such as polypropylene and polyethylene. PHAs are often considered a model for biologically sourced polyesters due to their chemical tunability, benign (non-toxic) degradation byproducts, and robust biocompatibility [27,59]. Although PHAs are produced through purely microbial synthesis, their large-scale commercialization is hindered by the lack of environmentally friendly, cost-effective downstream recovery methods, which can make them less competitive than chemically synthesized polyesters [60].

PHAs can biodegrade effectively without specific temperature requirements, similar to other biopolymers, in landfills and in environments unfavorable to degradation, such as freshwater and marine ecosystems [61,62]. However, PHAs have slower degradation kinetics than other biopolymers, such as cellulose, which can affect their potential as a sustainable material. A common strategy to increase the degradation rate of PHAs is to blend them with cellulose, which enhances water accessibility and facilitates enzymatic degradation [63].

Beyond environmental sustainability, their favorable biocompatibility and non-toxicity make PHAs promising candidates for biomedical applications, particularly in tissue engineering [34]. This explains the recent surge of interest in research on the biomedical applications of PHAs in regenerative engineering, with a strong emphasis on bone regeneration [35].

Among PHAs, poly(3-hydroxybutyrate) (PHB), a natural polymer typically derived from specific bacterial species, receives significant attention. It is an eco-friendly biopolymer that can be obtained from low-cost, renewable feedstocks and can be degraded both aerobically and anaerobically without the release of harmful byproducts [27].

Beyond the simple homopolymers, PHAs copolymers are attracting interest for their tunable properties. By altering the composition of their comonomer, their thermal and mechanical properties can be tailored [35]. A copolymer of economic significance, poly(3-hydroxybutyrate-co-3-hydroxyvalerate (PHBV), is well known for its versatility. PHBV is a high-crystalline copolymer of 3-hydroxybutyrate (3HB) and 3-hydroxyvalerate (3HV). Its impact strength increases with an increase in its 3HV content. However, higher 3HV levels reduce crystallinity, tensile strength, melting points (Tm), and the glass transition temperature (Tg) [64,65] Notably, PHBV exhibits superior flexibility to PHB, although it has been shown that its composites with PLA improved its stiffness property, comparable to poly(ethylene terephthalate) (PET) [66]. Therefore, manipulating the ratio of 3HB and 3HV monomers or blending PHBV with other biopolymers can enable the development of PHBV materials with desired properties, ranging from more ductile films to stiffer molded objects, thereby expanding their application range [34,66,67].

Nevertheless, Jin et al. (2023) noted that commercially available PHBV has a lower 3HV content, which confers brittleness and limits broader use [35]. To address this issue, several studies have shown that blending PHBV with other polymers, particularly PCL, PLA, PBAT, and polybutylene succinate (PBS), produces composites with targeted properties for environmental, packaging, and biomedical applications [35].

- Chitosan

Chitosan is a non-synthetic, biocompatible biopolymer that is often considered the second-most-abundant natural biopolymer after cellulose. Chitosan mainly originates from the deacetylation of chitin, the structural component that forms the exoskeleton of many invertebrate organisms. The deacetylation process of chitin typically occurs in a strong alkaline environment, although it can also be achieved through enzymatic hydrolysis [68].

Based on structural similarities, chitosan is closely related to cellulose and chitin, and it stands out as the only known cationic polysaccharide. It consists mainly of β-(1-4) linked glucosamine units, with a variable proportion of N-acetylglucosamine residues. Unlike cellulose, the cationic character of chitosan allows its electrostatic interaction with other negatively charged biomolecules. These properties not only broaden chitosan’s biomedical applications but also render its behavior strictly pH-dependent, which can be harnessed for the design of chitosan-based materials [36,69].

The presence of amino and hydroxyl groups in chitosan confers it with exceptional biological functionalities and chemical properties. Chitosan is relatively soluble in various media, interacts with microbial cell walls, exhibits viscosity, film-forming capacity, and has unique optical properties [36]. In biomedical research and applications, chitosan has been extensively studied for use in tissue engineering. The biomedical application of chitosan results from its consideration as a biopolymer that is non-toxic, biodegradable, biocompatible, and exhibits antimicrobial properties [7]. However, a practical challenge with chitosan is its limited solubility in body fluids, which restricts its broad application for drug delivery [70]. One key strategy to address chitosan’s limited solubility is to blend it with hydrophilic plasticizers to enhance its moisture-absorption capacity [37].

In the context of sustainability, chitosan has also received growing interest for use in sustainable food packaging, mainly due to its multifunctional properties. Its intrinsic antimicrobial properties, combined with its barrier properties, make it a promising material for biodegradable food packaging films. Chitosan can also be particularly useful when combined with several additives, particularly bioactive compounds, polymers, and nanoparticles, which enhance its thermal, mechanical, and functional properties. Although the addition of plasticizers (such as PEG and glycerol) can improve the mechanical properties of chitosan composite films, the hydrophilicity of these plasticizers often results in increased moisture absorption, thereby compromising the chitosan barrier property [37].

Nevertheless, chitosan composite films can represent a biodegradable alternative to non-degradable petroleum-based plastics for packaging. They have been shown to have the ability to preserve perishable foods, including meat, dairy products, and seafood, thereby reducing spoilage and extending their shelf life [71].

In addition to food packaging, chitosan-based bionanocomposites are emerging as a cost-effective and highly efficient adsorbent for removing highly toxic contaminants in wastewater purification. However, the adsorption efficiency largely depends on the type of grafted solid materials on the chitosan composite, notably activated carbon, clays, and metals [72]. These studies bolstered the versatility of chitosan and chitosan-based materials for applications in biomedicine, packaging, and environmental sustainability.

- Silk Protein

Silk Protein is a natural polymeric material primarily produced by certain members of the phylum *Arthropoda*, including silkworms, spiders, fleas, and mites [39]. Although silk protein has received less attention than other biocompatible polymers, its favorable mechanical properties and controlled degradability have sustained scientific interest in its biomedical applications [40].

Silk possesses distinctive properties that continue to attract scientific interest. Its composition, structure, and properties differ depending on the source species, resulting in variations in environmental adaptability and functional roles [41]. Variation in the sequence of amino acids in the silk protein produced by different silkworm species results in distinct chemical, biological, and physical properties [73]. Among these species, *Bombyx mori* silk protein stands out for its excellent properties and is the most widely studied for biomedical applications [74].

Silk protein is widely considered a biocompatible and less immunogenic material with impressive mechanical properties. These properties have supported its growing applications in biomedicine. Silk protein has been applied particularly in the development of nanospheres, microspheres, and membranes for therapeutic purposes [75]. However, silk protein (specifically silk fibroin) has a relatively slow degradation rate, which may negatively impact some biological applications. To address this issue, silk fibroin is enzymatically cross-linked with genipin or other materials to modify its stiffness, making it customizable for specific biological applications [40].

Recent studies have highlighted how the two main components of silk protein (sericin and fibroin), when combined with nanoplatforms, are emerging as effective materials for wound repair [76]. Another area of growing interest is the development of silk protein-based hydrogels, as they integrate well into tissue, exhibit lower immunogenicity, and are degradable, making them suitable candidates for wound dressings [77].

Silk protein-based composites are also being increasingly explored for their strong potential in bone tissue repair and regeneration. These silk-based protein composites are reported to exhibit favorable physicochemical properties and osteogenic signaling capabilities. This makes silk protein-based composites promising in bone tissue regeneration [78]. Despite the favorable mechanical and biocompatible properties of silk protein, the rearing of its insect producers requires the use of non-renewable energy sources, which has a negative environmental impact [79].

Apart from biomedical uses, silk protein is also being explored as a green and sustainable material. Silk protein has expanded potential because it can be used alone or in combination with other polymers, thereby broadening its potential as an advanced material [80]. The incorporation of low-cost polymers, particularly cellulose or starch, into silk protein composites may not only enhance their economic feasibility but also modify their degradability and flexibility for applications in various fields.

- Collagen

Collagen represents a principal fibrous structural protein often considered the most highly distributed and abundant protein in the animal body. It has impressive mechanical properties, which are strongly influenced by its triple-helical structure, composed of intertwined polypeptide chains. Each of the polypeptide chains is composed of a unique repeating sequence of glycine, proline, and hydroxyproline amino acids, which reinforces the helical structure of the protein. These properties impart collagen with strength and resilience, allowing it to perform structural functions in various tissues of the body. In addition to its structural properties, collagen is inherently biocompatible and low-immunogenic, increasing its potential breadth for biomedical applications.

However, gels derived from native collagen (especially marine) often exhibit insufficient mechanical properties for certain load-bearing applications [45]. Like other natural polymers with low mechanical properties, improved collagen mechanical properties can be achieved with crosslinking with chemical agents. However, the use of a chemical crosslinking agent, such as formaldehyde or glutaraldehyde, may induce toxicity [45].

Collagen can be obtained from various sources, each with its own advantages and disadvantages. Collagen sourced from the Achilles tendon, pig, and sheep skin raises concerns about pathogen transmission and immunogenicity, which necessitate the exploration of safer alternatives, such as marine and recombinant collagen [42,43,46].

Some of the advantages of marine collagen over mammalian collagens include simpler extraction methods, lower denaturation temperatures, minimal inflammatory response, and overall safety. However, marine collagen is also limited by variability in structure owing to differences in source species, environmental conditions, and other factors. Such differences make it difficult for batch standardization and reproducibility, which are necessary for regulatory approval in biomedical applications [45].

Still, collagen and its derivatives are being developed in various forms, including hydrogels, scaffolds, sponges, and thin films for applications in tissue engineering, wound healing, regenerative medicine, and drug delivery [81]. For example, collagen-based nanofibers, owing to their bioactivity and structural similarities with the extracellular matrix, provide essential support for wound repair by stimulating cell adhesion, proliferation, migration, and differentiation.

Outside the biomedical field, collagen is also explored as a sustainable, environmentally friendly material for food packaging and other environmental applications. A recent study on collagen fiber film (CFF) derived from *Halocynthia roretzi* reported good tensile, water resistance, and biodegradability properties, coupled with a positive preservation effect on strawberries and pork when combined with chitosan [47].

Collagen packaging films have relatively favorable mechanical properties compared to other biopolymers, owing to collagen’s unique triple-helical structure, which influences the film density. Despite these potentials, the broad application of collagen-based films, especially those derived from safer marine collagen, is limited by the brittleness of collagen, its high water solubility, and high production costs [45]. A more effective approach to addressing this issue could be to explore highly thermally stable collagen sourced from marine species, as chemical crosslinking may increase toxicity.

- Alginate

Alginate is a natural anionic, linear biopolymer commonly obtained from brown algae (such as bladderwrack, kelp, and sargassum) and certain bacteria (such as Pseudomonas aeruginosa). Structurally, alginate consists of α-mannuronic acid (M) and β-guluronic acid (G) linked by 1,4-glycosidic bonds. Alginate physicochemical properties are largely determined by its composition and the G-to-M ratio, which is primarily determined by the source species and geographical origin. Alginate has been explored for various human applications due to its favorable properties [82]. However, reproducibility and standardization remain challenging, as variability in alginate composition is influenced by factors such as species, season, and geographical origin [82].

Alginate exhibits beneficial properties, including biodegradability, biocompatibility, ease of gelation, non-toxicity, and high water absorption, rendering it a promising material for various biomedical uses [82]. For example, the biocompatibility and water retention ability of alginate-based microcapsules or nanoparticles offer them the ability to protect the drug constituent from gastric acid damage through controlled release, resulting in a better absorption rate [83,84]. However, alginate hydrogels exhibit poor mechanical integrity, and under physiological conditions, their stability can be compromised by their insufficient moisture barrier properties [85].

In wound dressings, alginate-based hydrogels exhibit good biocompatibility and are associated with minimal inflammatory reactions in the targeted areas of bone and joint injuries [86]. However, due to its high moisture absorbance capacity, an alginate/chitosan composite is typically developed not only to reduce moisture permeability but also to bestow it with additional antimicrobial and enhanced mechanical properties [82].

From sustainability perspectives, the multifaceted properties of alginate have gained growing scientific interest for its consideration in sustainable food packaging. For example, alginate-based films can prolong the freshness and reduce the spoilage of perishable foods such as meats, fruits, and vegetables. These films function by inhibiting microbial growth while maintaining the oxygen level and moisture content of the preserved foods. Based on these combined effects, alginate-based films can maintain food quality and offer a promising, eco-friendly alternative to conventional plastic packaging [85]. Despite the progress made with regard to alginate-based packaging films, certain challenges remain for practical applications. These include large moisture absorption, which can impair food quality and the mechanical integrity of the film, as well as difficulty in scaling up packaging film production [85].

#### 2.2.2. Synthetic Polymers

- Polylactic acid (PLA)

Polylactic acid (PLA) is widely recognized for its distinct advantage of being synthesized from renewable materials, such as rice bran, corn, and potato starch [27]. It is a linear aliphatic polyester chain that can be synthesized through the polycondensation or ring-opening polymerization of lactic acid monomers [55].

PLA is extensively used in the development of medical devices and sustainable packaging, owing to its well-known biocompatibility and biodegradability [27,55]. However, the inherent stiffness of PLA, which can make it unsuitable for certain load-bearing applications, necessitates blending it with other polymers to enhance its performance [19].

**Table 2:** Comparison of key properties of synthetic polymers.

Polymer	Mechanical Strength	Degradation Rate	Biocompatibility and Biodegradability	Key Limitations	References
Polylactic acid (PLA)	Satisfactory; depends on stereoisomer distribution.	Accelerated at high temperature and humidity	Biocompatible and biodegradable	Weak mechanical properties	[11]
Polyglycolic acid (PGA)	~31 MPa (pure PGA), 4–5% elongation	90% mass loss in 20 days (70 °C, water)	Biocompatible and biodegradable	High crystallinity leads to low elongation (≈4–5%), limiting flexibility.	[27,87]
Poly (lactide-co-glycolide) (PLGA)	High (up to 2× with HA/β-TCP); load bearing	Tunable (1–6 months)	Biocompatible and biodegradable	Burst release, acidic microenvironment, brittleness	[10,20,54]
Polycaprolactone (PCL)	~23 MPa (bulk), 2.5 MPa (electrospun), ~700% elongation	4.8% mass loss (32 weeks), full resorption ≈14 months	Biocompatible and biodegradable	Slow degradation and hydrophobicity	[27,88,89,90]
Polyethylene glycol (PEG)	Flexible and elastic, 1.24–1.44 Mpa (PVA-PEG-CNF hydrogel)	Very slow; partial degradation in blends after 35–45 days	Biocompatible	Poor biodegradability, moisture instability, and MW variability	[91,92]
Polyurethane (PU)	10–40 MPa (bio-based PU); up to 1000% elongation	Weeks to months (depending on structure and environment)	Biocompatible and biodegradable	Isocyanate toxicity, poor biodegradability, and recycling limits	[93,94,95]
Polyvinyl Alcohol	High (adjustable via crosslinking and FT cycles)	Weeks–months; accelerated by ester copolymerization	Biocompatible	Non-degradability, swelling, process sensitivity	[96,97,98]

Therefore, in addition to its intrinsic properties, PLA is commonly improved for several applications. For example, the mechanical and degradation properties of PLA were tuned in a 3D-printed PLA/PCL scaffold by varying the PLA/PCL ratio, providing invaluable insights for the development of custom scaffolds to meet specific needs. However, for such blends to be put to practical use, they may need to be optimized for a specific application. For example, an increased PLA content in the blend may make the composite more appropriate in applications where rapid degradation is necessary (e.g., soft tissue implants). On the other hand, a load-bearing application with the PLA/PCL blend scaffold will require a higher PCL content for long-term structural support [19].

In another study, PLA’s tensile and thermal performance has been shown to improve significantly through filler hybridization derived from rice husk and biocarbon, resulting in a composite suitable for structural applications and enhancing sustainability. However, a high concentration of the biocarbon results in poor matrix interaction, signifying a challenge that needs to be addressed for large-scale manufacturing [99].

In addition, a compostable composite based on a PLA/polybutylene adipate-co-terephthalate (PBAT) blend with ε-poly-L-lysine (ε-PL) was developed to impart antimicrobial properties, yielding an antimicrobial packaging film that enhances food safety and increases shelf life. Despite the impressive advances, the study highlighted impairments in the mechanical and moisture-barrier properties of the composite due to the hydrophilic nature of the ε-PL, which decreases the crystallinity of the material [100]. Therefore, a critical challenge remains in developing PLA-based composites with suitable antimicrobial properties without compromising their mechanical or barrier properties.

- Polyglycolic acid (PGA)

Polyglycolic acid (PGA) is a synthetic, thermoplastic, biodegradable polymer based on a linear aliphatic polyester. PGA is synthesized by the polymerization of ring-opening of cyclic glycolide monomers [101]. The two-step process of PGA degradation involves the initial penetration of water into the amorphous region, where ester bonds break, followed by the hydrolytic cleavage of the crystalline regions [102].

Despite the biodegradability of PGA and its structural similarities to PLA, it is known for its favorable barrier properties, high mechanical properties, and heat resistance compared to PLA [103].

Consequently, PGA has been applied in various fields of biomedicine, including drug delivery, tissue engineering, and biomedical devices [55]. Despite its potential, PGA has disadvantages, including rapid hydrolytic degradation and poor toughness, which can limit its long-term mechanical stability. As such, PGA is blended with other polymers to enhance its mechanical properties. The water resistance and mechanical properties of PGA can also be enhanced through graft or chain-extension reactions with chemicals such as glycidyl methacrylate [103].

Beyond biomedicine, a PBAT/PGA blend film exhibits enhanced mechanical properties and biodegradability in ambient environments, highlighting the potential of PBAT/PGA films as a sustainable material for packaging applications. However, a thorough investigation of the performance of PBAT/PGA under different environmental conditions and an evaluation of the migration behavior of the PBAT/PGA component under various conditions are necessary before practical packaging applications.

- Poly (lactide-co-glycolide) (PLGA)

Poly (lactide-co-glycolate) (PLGA) is synthesized by the copolymerization of glycolic acid (GA) monomers with L-lactide and LD-lactide (LA). Its physical form, whether largely amorphous or more crystalline, depends on the molar ratio of lactide to glycolide in the copolymer. The variation in this ratio also governs the degradation rate [104]. The adjustment of these proportions often provides an opportunity for fine-tuning the chemical and mechanical properties of PLGA for specific applications [54].

PLGA is a crucial biopolymer in drug delivery and regenerative medicine. It has increasingly been considered for these purposes owing to its tunable degradation properties, biocompatibility, and ability to sustain and localize drug release. However, for orthopedic or certain applications that require mechanical and thermal stability, PLGA is typically reinforced or grafted with other chemicals. Some key PLGA reinforcement agents include hydroxyapatite and β-tricalcium phosphate (β-TCP), which enhance its structural integrity in a wet environment and improve mechanical strength. [20].

A typical example of a sustained-release study is one in which a peptide-loaded PLGA microsphere exhibits an enhanced release profile and significantly reduced burst release. This provides insight into the efficient preparation of microspheres for drug delivery using a microfluidic device [105]. Although such studies may serve as a benchmark for preparing peptide-loaded microspheres, rigorous optimization studies are needed to achieve consistent encapsulation efficiency and reproducible performance under physiological conditions before clinical translation.

- Polycaprolactone (PCL)

Polycaprolactone (PCL) is a synthetic, biodegradable, linear aliphatic polyester produced by the ring-opening polymerization of caprolactone monomers in the presence of a metal anion catalyst. Its tensile stress ranges from 12 to 30 MPa, with elongation at break between 400% and 900% [106].

The melting point of PCL ranges from 58 to 60 °C. Some properties that support its biomedical applications include its low cost, high toughness, and solubility in most organic solvents. Although both aerobic and anaerobic microorganisms can degrade PCL, the crystallinity and molecular weight of PCL may influence the rate of degradation. In addition, its poor biological activity due to its hydrophobic structure and slow degradation rates are factors that could limit its extensive applications [27].

The complete degradation of PCL can take 2 to 3 years, making it unsuitable for applications that require rapid degradation, particularly in drug delivery. The degradability issue of PCL can be addressed by blending it with degradable materials, particularly PLA and PGA, thereby reducing its crystallinity. PCL can also be blended with biological materials, particularly collagen and hydroxyapatite, to enhance its biological properties for specific applications, such as craniofacial tissue regeneration [89].

A growing number of studies have shown that PCL properties can be improved by producing its composites with other polymers for specific applications. For instance, a new biodegradable and biocompatible PCL-PU-semi-IPNs scaffold was developed, supporting the attachments and proliferation of cells, highlighting an advanced material for skin tissue engineering applications.

In another study, electrospun PCL-based membranes were developed, demonstrating promising properties for localized drug delivery, tissue regeneration, with mechanical properties comparable to those of commercially available guided bone regeneration (GBR) and guided tissue regeneration (GTR) membranes. Although these studies are promising, there are still biocompatibility and safety concerns, especially regarding degradation products that may stimulate an inflammatory response. Additional in vivo studies are also needed to assess the clinical applicability, biocompatibility, long-term stability, and consistent therapeutic potential of the incorporated agents before practical applications [90].

- Polyethylene Glycol (PEG)

Polyethylene Glycol (PEG) is a synthetic and hydrophilic polymeric material widely recognized for its versatility. It can also be distinguished by its several end functional groups and varying chain lengths [107]. PEG can be synthesized either through the polycondensation polymerization of ethylene glycol or through the ring-opening polymerization of ethylene oxide [91].

Some of the distinct physicochemical properties of PEG, particularly its good water solubility, inherent biocompatibility, and tunable molecular structure, have placed it as one of the most widely applied synthetic polymers across pharmaceuticals, biomedicine, and related fields. In addition, PEG can be customized through molecular modifications and polymer design to suit specific applications in tissue engineering, drug delivery, and food packaging. Despite the enormous potential of PEG, its broad biomedical application is limited by its potential toxicity and limited biodegradability [91].

An expanding area of PEG application in biomedicine is the development of PEG-based hydrogels and drug delivery technologies. PEG-based hydrogels continue to be applied for the treatment of various wound injuries due to their responsiveness to environmental stimuli and controlled drug release to a specific target area, thereby minimizing off-target effects and enhancing the success of chemotherapy [108].

In the context of food packaging and preservation, PEG-based nanocomposite films combined with antimicrobials have been shown to improve food quality, thereby mitigating spoilage and extending shelf life. However, despite PEG’s benefits, its hydrophilicity could, in the long term, affect food quality and the mechanical integrity of packaging films. Additionally, strict consideration of dosage is necessary for its safe use [91]. Nevertheless, these advances underscore the potential of PEG-based materials not only in biomedicine but also in the production of food packaging and solutions.

- Polyurethane (PU)

Polyurethane (PU) is an important synthetic polymeric material, mainly distinguished by its urethane bonds. The synthesis of PU occurs through the process of addition reactions between isocyanates and alcohols [94]. Based on its structural composition, PU is divided into hard and soft segments. The hard segments are typically composed of isocyanates and chain extenders, while the soft segments are derived from polyols. By adjusting the ratio, structure, and molecular distribution of these segments, PU can be engineered into functional materials with antibacterial, self-healing, anti-aging, and anticoagulating properties [93].

The growing interest in the use of PU in biomedical applications can be associated with its low cytotoxicity, inherent chemical stability, and biocompatibility. Recent reviews, especially the work of Cui et al. (2023) [93], have comprehensively emphasized the growing interest in the application of PU in orthopedics, biosensors, wound dressings, and cardiovascular applications, reflecting the growing importance of PU in biomedical innovations. However, an active area of research is ensuring PU stability to prevent undesired degradation and aging for long-term applications. Also, for short-term applications, efforts are being made to tailor the PU degradation rate so that it does not degrade too fast, releasing a product that may stimulate inflammatory reactions, or too slow, delaying patient recovery and causing undesired damage [93].

From a sustainability perspective, efforts have been focused on bio-based PU materials derived from renewable sources, such as vegetable oils, polysaccharides, and lignin, to reduce the environmental challenges posed by PU production and may open new avenues for its commercialization. Although bio-based PU materials offer numerous advantages, achieving PU materials with satisfactory mechanical properties for the desired application is still challenging. Efforts have been made to reinforce PU-based materials with fillers, especially chitosan, to produce bio-composites with suitable properties for long-term use. Still, scalability and economic feasibility continue to limit large-scale production of PU-based materials [95].

- Polyvinyl Alcohol (PVA)

Polyvinyl Alcohol (PVA) is a biodegradable polymeric material that has attracted considerable attention in recent years. The strong interest in PVA stems from its favorable properties, including hydrophilicity, biocompatibility, non-toxicity, and mechanical performance. PVA is mainly synthesized through the hydrolysis of poly(vinyl acetate) (PVAc), which is formed via the polymerization of vinyl acetate monomers [97].

PVA has been considered for many biomedical applications. However, it is particularly valuable in the production of PVA-based hydrogels. The strong interest in PVA-based hydrogels stems from their combination of favorable properties, including good mechanical strength, suitable water content, and excellent biocompatibility. However, when specific mechanical properties are desired, such as in tissue engineering, filtration, or membrane production, the inherent mechanical properties of PVA are often insufficient and have to be improved through various crosslinking strategies [96].

Consequently, with advances in synthesis and crosslinking strategies, PVA-based materials, such as PVA-based hydrogels, have the potential to become promising materials for various biomedical applications. Advanced cross-linking strategies using cross-linking agents often yield PVA with enhanced flexibility, cytocompatibility, and antimicrobial properties, thereby expanding their potential applications in areas such as drug delivery, regenerative medicine, contact lenses, and related biomedical fields. On the other hand, the improved mechanical and thermal properties achieved by crosslinking often reduce the PVA biodegradation rate, a drawback which is particularly undesirable in drug delivery systems. As a result, even with significant progress in developing PVA materials, particularly PVA-based hydrogels, they have not yet reached an appropriate level for large-scale production [96,109].

In the context of environmental sustainability and green packaging, PVA is also receiving considerable attention as a green packaging material. Trigui et al. (2025) developed a PVA-based packaging film reinforced with nanofibril fillers, demonstrating improved barrier performance, thermomechanical stability, and mechanical strength, highlighting the potential of PVA-based materials as an environmentally friendly packaging alternative [110]. However, further optimization of the coating process on both plastic and paper packaging films is still needed before practical applications [110].

- Glycerol-based polyesters

Glycerol-based polyesters have emerged as a promising class of biodegradable and biocompatible materials, particularly for biomedical applications. These polymers are typically synthesized via polycondensation of glycerol with non-toxic dicarboxylic acids, producing materials with tunable mechanical and degradation properties. Among these, poly(glycerol sebacate) (PGS) is the most extensively studied, followed by poly(glycerol succinate) (PGSu), which has shown emerging potential in related fields.

Poly(glycerol sebacate) (PGS) was first reported in 2002 in the context of tissue engineering, as a tough biodegradable polyester synthesized for soft tissue engineering [111,112]. PGS is relatively inexpensive, exhibits thermoset elastomeric properties, and is bioresorbable, meaning it degrades in vivo, with its degradation products eliminated via natural metabolic pathways. Moreover, its mechanical properties and degradation rates can be tailored to meet specific application requirements [113].

The common starting materials for PGS synthesis are glycerol and sebacic acid. Glycerol is a basic building block of lipids, while sebacic acid, a natural metabolic intermediate in the ω-oxidation of medium- to long-chain fatty acids, is chosen for its favorable toxicological and polymer chemistry profile. Both monomers are considered safe: the U.S. Food and Drug Administration (FDA) has approved glycerol for use as a humectant in foods, and polymers containing sebacic acid are also considered safe [114].

The synthesis of PGS is guided by five key criteria based on its intended use: Hydrolytic degradation is preferred to reduce variability from enzyme-based degradation, and ester bonds are included to enable hydrolysis. The polymer should have a low degree of cross-linking, which must be hydrolysable and chemically identical to the backbone to ensure uniform degradation. The starting materials must be non-toxic, with at least one being trifunctional and one supplying hydroxyl groups for hydrogen bonding (as mentioned above, the starting materials glycerol and sebacic acid meet this criterion) [115]. The conventional method for synthesizing PGS, i.e., polycondensation synthesis, involves a two-step procedure that incorporates a pre-polymerization step to form low-molecular-weight polymers/oligomers, followed by a curing step to cross-link these products and shape the final material [116,117]. Alternative synthesis methods have also been explored, including microwave-assisted processes, catalytic or enzymatic synthesis (e.g., using lipase), photopolymerization, and urethane cross-linking [118,119].

PGS is characterized as a transparent, nearly colorless polyester with mechanical behavior similar to soft biological tissues such as the cornea, arteries, spinal cord, and some muscle types. Structurally, hydroxyl groups on the carbon backbone enhance the hydrophilicity of the polymer [120]. PGS exhibits nonlinear stress–strain behavior, typical of soft elastomeric materials. As a partially semicrystalline polymer, its thermal properties are influenced by both its glass transition temperature (Tg) and melting temperature (Tm). PGS remains stable up to 250 °C and exhibits a single weight loss step between 320 °C and 475 °C [121].

Poly(glycerol succinate) (PGSu) is another glycerol-based polyester that can be synthesized via the polycondensation of glycerol with succinic acid, a dicarboxylic acid similar to adipic and sebacic acids [121]. Succinic acid is derived from the fermentation of biomass and is now produced at an industrial scale. The synthesis of PGSu typically involves bulk polycondensation in the absence of solvent and catalyst, without generating toxic byproducts [122]. PGSu macromolecules can adopt various architectures, including dendrimers, branched, highly branched, and hyperbranched polymers. The synthesis conditions significantly affect the physical and mechanical properties of hyperbranched polyesters (HBPEs), due to variations in molecular weight, cross-linking density, and degree of branching. However, compared to PGS, there is currently limited data available on the mechanical properties of PGSu [123].

Poly(glycerol succinate) has been used as an accelerant for poly(caprolactone) degradation, promoting moisture penetration into hydrophobic matrices. Furthermore, hydrophilic PGSu oligomers can be functionalized with fatty alkyl chains to form amphiphilic structures, which may serve as bio-based surfactants in various personal care and household products, such as shampoos, body washes, and kitchen cleaners. These bio-based surfactants could reduce the skin and hair damage associated with synthetic detergents [124]. PGSu shares similarities with PGS, particularly in its potential for tissue engineering applications. PGSu has been proposed as a scaffold material or scaffold additive for cardiac, bone, cartilage, nerve, and corneal tissue regeneration [125].

Despite the many advantageous properties of glycerol-based polymers, continued research is needed to enhance their performance and functionality for clinical use. Further development and modification strategies are essential to fully realize their potential, especially in the regeneration of damaged tissues and the treatment of various diseases.

## 3. Recent Technological Advancements

### 3.1. Surface Modification and Functionalization

A significant drawback of many inexpensive polymers is their resistance to various chemical treatments, which is a result of their naturally low surface energy. Even though their bulk properties, including density, chemical resistance, and mechanical flexibility or rigidity, may satisfy the needs for different applications, their surface qualities frequently fall short [126]. This concern is especially significant in biomedical applications, where materials need to interact well with water-based environments. For example, since biological processes take place in liquid mediums, the polymers used in these applications must demonstrate adequate wettability. If this property is lacking, biological fluids like blood can form droplets and slide off the surface, which obstructs proper functionality. Therefore, polymer-based biomedical devices intended for handling liquids must undergo surface treatment or functionalization to attain the required wettable properties [127].

Currently, conventional techniques for altering the surfaces of polymeric substrates involve either attaching polyethylene glycol chains to the polymers, particularly those used in drug and gene delivery, or chemically treating the polymer to introduce functional groups, nanoparticles, and other polymers (both natural and synthetic) [128]. Conversely, methods based on plasma and irradiation can enhance the chemistry and physical characteristics of the BC in a single step while preserving its inherent bulk properties. We will elaborate on each of these techniques in more detail in this section.

#### 3.1.1. PEGylation

PEGylation is the process of attaching polyethylene glycol (PEG) chains to various biomolecules, including hydrophobic polymers, drugs, nanoparticles, proteins, and peptides, to improve their therapeutic effectiveness while minimizing the associated toxicity [129]. The idea of PEGylation was first introduced by Davis and Abuchowski in 1970, when they successfully altered albumin and catalase using PEG. Since then, the technology has been extensively advanced and broadly utilized by various biomedical research and therapeutics [130]. PEG can be applied to nanoparticle (NP) surfaces using three primary strategies, namely covalent grafting to form a stable chemical bond, physical adsorption through electrostatic or hydrophobic interactions, and conjugation with hydrophobic molecules to create macromolecules that self-assemble with other compounds, resulting in PEGylated NPs in solution [131].

PEGylation has been widely utilized in biomedical applications to improve the stability and performance of nanomaterials [132]. For instance, PEGylation has been demonstrated to enhance the stability of micelles, liposomes, dendrimers, gold nanoshells, quantum dots, and polymeric nanoparticles (NPs) in vivo, thereby improving therapeutic efficacy [133]. PEG-modified NPs exhibit increased hydrophilicity and near-neutral zeta potential, reducing protein adsorption (opsonization) and subsequent clearance by the mononuclear phagocyte system [134]. Additionally, the hydrated PEG chains increase the hydrodynamic size of NPs, providing steric shielding that reduces renal filtration and limits degradation by enzymes and antibodies. This prolongs circulation time and supports more efficient, localized drug release [135].

To date, over 20 PEGylated liposomal or RNA-based formulations have received approval from the United States Food and Drug Administration, including Doxil^®^ and Macugen^®^, which are employed in the treatment of cancer and neovascular age-related macular degeneration, respectively [136,137]. Beyond drug delivery, PEGylation also enhances the stability and biocompatibility of nanomaterials such as graphene, without compromising their intrinsic properties [136,137,138,139]. For instance, PEG coatings have been shown to improve the solubility and stability of graphene and extend the circulation time of PEGylated PLGA nanoparticles by reducing their immunogenicity and resistance to immune clearance [140].

#### 3.1.2. Chemical and Plasma Treatments

- Chemical treatments

The chemical modification of polymer surfaces is an important area where traditional physical methods fall short or are unsuitable for industrial needs, particularly in biomedical applications, where improved properties are necessary without changing the surface texture. Many chemical surface treatment techniques utilize wet processes, where the polymer is submerged or coated/sprayed with a solution to improve its surface characteristics and eliminate debris and microbes, creating a sterile environment appropriate for biomedical use [141]. Chemical treatments rely on the reactive sites or functional groups found on polymer chains. Typically, these processes are conducted in a watery environment (wet chemical etching), which enhances the number of functional groups on the surface. These functional groups are then activated and later interact with other molecules present in the solution [127].

Although wet chemical treatments offer a diverse array of reagents for selectively treating polymers at scale and low cost, they necessitate a careful approach, since the reaction rate is influenced by the reagent’s strength, the composition of the material, and the duration of treatment. Furthermore, additional steps such as rinsing, washing, and drying are necessary before any further processing of the polymer, which inevitably leads to an increase in the amount of hazardous waste produced during a single surface treatment operation. Consequently, surface modification via wet chemical methods is advantageous if the application is non-invasive and poses minimal side effects, with proactive etching and alterations in the material’s bulk crystalline phase being of lesser significance for the intended application. For instance, biomedical implants demand both elasticity and strength alongside surface modifications to enhance functionality and affect bio-interactions with surfaces [141].

Various approaches for altering the chemistry of polymers include substituting the existing functional groups on the polymer, such as replacing the hydroxyl groups in the polymer with different functional groups [142,143], crosslinking with other polymer materials through either covalent or non-covalent bonds [144], or creating physically attached composites with polymers that exhibit improved characteristics [145,146]. The creation of composites involving metal or metal chalcogenide nanomaterials, along with the integration of carbon-based nanocomposites, has been well established [145]. The key benefit of developing nanocomposites by incorporating metals or metal chalcogenides is that the resulting polymer can merge the properties of multiple materials, thereby enhancing both the mechanical characteristics and functionalities beyond those of the individual composites alone. Although chemical treatment is an easy way to render a polymer wettable, this process is declining due to the ecological impact caused by the use of excessive organic solvents and other toxic chemicals [2].

- Plasma treatment

Plasma is a partially ionized gas composed of free electrons, ions, radicals, and neutral molecules or atoms. It can be generated through several techniques, including direct current (DC), alternating current (AC), and radiofrequency (RF) discharge. In these processes, gases are excited into energetic states by means of radio-frequency waves, microwaves, or electrons emitted from a heated filament discharge [147]. The plasmas most commonly used for surface treatment are cold non-equilibrium plasmas, in which the electrons possess much higher translational energies (1–10 eV) than the ions, molecules, or radicals (around 0.025 eV, corresponding to 298 K). Only a small fraction of the gas molecules are ionized under these conditions. Low-pressure plasmas, typically operated at 0.1–10 Pa, are especially effective for such applications, as the reduced pressure allows the plasma to penetrate more deeply into materials while minimizing surface damage [148].

In low-pressure plasmas, particles exhibit a longer mean free path, allowing them to travel greater distances before colliding with gas molecules or surfaces. As a result, the plasma can penetrate more effectively and interact with materials at deeper levels, leading to enhanced surface modifications [126]. For large-scale surface modifications, atmospheric-pressure plasmas are often preferred because they operate at or near room temperature and do not require a vacuum system. These plasmas are sustained by gas mixtures capable of maintaining a discharge at atmospheric pressure, eliminating the need for costly vacuum chambers. This makes it possible to treat extensive surface areas efficiently, thereby overcoming a key limitation of vacuum-dependent surface modification processes in industrial applications [149].

Plasma treatment provides enhanced flexibility and typically utilizes environmentally friendly substances such as noble gases, oxygen, and nitrogen, along with minimal amounts of organic precursors, and requires relatively straightforward equipment, which includes a vacuum chamber, a plasma source to create the discharge, gas supplies to fuel the plasma, and vacuum equipment like vacuum pumps [150]. When a surface is exposed to plasma, two primary types of modifications can occur. (i) Functionalization by reactive species: inert gases activated in the plasma can generate reactive species that interact with the polymer surface. For example, oxygen plasma can introduce hydroxyl groups through surface hydroxylation. (ii) Deposition of polymeric films: when organic molecules such as saturated or unsaturated hydrocarbons are used to generate plasma, thin polymeric coatings can be deposited onto the surface [127].

The effect of plasma treatment on surface characteristics depends strongly on the type of gas and the plasma conditions. Oxygen plasma introduces carboxyl and hydroxyl groups, while hydrogen plasma primarily yields hydroxyl groups through single-bond formation. Ammonia and nitrogen plasmas generate nitrogen-containing functional groups on the surface, whereas water plasma treatment results in the incorporation of hydroxyl groups [151]. Although plasma treatment offers several benefits, the effects are typically temporary, causing the altered surfaces to slowly regain their original wetting and adhesion characteristics. Recent studies have shown that integrating plasma treatment with approaches such as PEG silane treatment can lead to a lasting hydrophilic modification and enhance the durability of the polymer surface [152].

### 3.2. Stimuli-Responsive Polymers

The ability to respond to external stimuli is a characteristic frequently observed in living organisms. Nature necessitates finely tuned assemblies and interfaces capable of reacting to changes in the environment to sustain biological processes. For instance, certain plants can fold their leaves to trap insects when touched, such as the Mimosa pudica and the Venus flytrap (Dionaea muscipula), which begins to close when its trigger hairs are mechanically stimulated [153]. Drawing inspiration from how nature responds and functions, scientists across the globe have been working on innovative functional materials to create actuators that can perform tasks in reaction to changes in their physical and chemical surroundings [154]. The capacity to tune polymer properties in response to external stimuli enables the design of intelligent materials with applications in controlled drug delivery, bio-separation, biosensing, biomimetic actuators, and immobilized biocatalysis, among others [155].

Stimuli-responsive polymers can be derived from either natural or synthetic sources or by integrating a responsive element or function into the existing structure of a polymer. Polymeric nanocarriers, particularly those designed for the delivery of bioactive substances, can be adapted into stimuli-responsive systems based on the mechanism of release triggered by specific stimuli, which can be either endogenous (such as pH, enzymes, temperature, redox potential, hypoxia, glucose concentration) or exogenous (including light, magnetism, ultrasound, or electrical impulses) to ensure effective biodistribution and controlled release of drugs or genes at designated locations [153,156].

#### 3.2.1. Temperature-Responsive Polymers

Temperature-responsive polymers, or thermoresponsive polymers, represent one of the most extensively studied classes of smart materials. They are generally categorized into two groups based on their temperature-dependent phase behavior: lower critical solution temperature (LCST) and upper critical solution temperature (UCST) types. The LCST corresponds to the temperature at which the binodal, or coexistence curve, reaches its minimum in the phase diagram, while the concentration at this minimum is referred to as the lower critical solution concentration (LCSC) [157]. Temperature-responsive polymers exhibiting a lower critical solution temperature (LCST) remain soluble in water (or organic solvents) when the temperature is below the LCST. Above this threshold, however, they become hydrophobic due to intensified hydrophobic interactions both within and between polymer chains. This reversible solubility transition has been widely exploited in diverse applications, including model protein design, triggers for self-assembly, “on–off” switches for protein functionality, cell sheet engineering, drug delivery carriers, chromatography, sensors, and adsorption materials [158].

The first polymer reported to exhibit LCST behavior was poly(N-isopropylacrylamide) (PNIPAM) in aqueous solution. Since its discovery, PNIPAM has become the most extensively studied thermoresponsive polymer, largely because its phase transition occurs near physiological conditions, at approximately 32 °C. This transition, which lies between room and body temperature, makes PNIPAM particularly attractive for biomedical applications [159]. To broaden the range of applications, several LCST-type water-soluble polymers have been identified [160]. Among these is Poly-N-vinylcaprolactam (PNVCL), a synthetic, non-ionic, water-soluble, thermoresponsive polymer that can shift from a liquid state to a gel state when the temperature exceeds its lower critical solution temperature (LCST), typically reported within the physiological range of 34–37 °C. In summary, PNVCL is an affordable and widely utilized polymer due to its low cytotoxicity, making it suitable for use in the biomedical sector for purposes such as wound healing, drug delivery, and tissue engineering [161]. Another example is poly(2-chloroethyl vinyl ether-alt-maleic anhydride), which displays LCST-type thermoresponsive phase behavior in selected organic solvents under mild conditions [162,163].

In contrast to LCST polymers, UCST polymers are soluble in solvents at temperatures above the UCST and become insoluble when the temperature drops below this threshold [164]. Specifically, increasing the temperature beyond the transition point leads to solubilization that is driven by enthalpy, as the interactions among polymer chains are diminished in favor of interactions between the polymer and the solvent [165]. This phenomenon arises due to particular interactions like hydrogen bonding and electrostatic interactions. The capability of UCST polymers to experience phase transitions triggered by temperature changes offers potential for a variety of applications, including insulating materials, catalysis, the assembly of nanomaterials, sensors, and protein separations [166]. At present, the most extensively studied UCST polymers include poly(sulfobetaine), poly(acrylic acid), and poly(acrylamide), all of which exhibit phase transitions in aqueous media under practical conditions [159].

Typically, temperature-sensitive homopolymers exhibit two main changes in properties: a shift from hydrophilicity to hydrophobicity, and the onset of aggregation/precipitation due to hydrophobic interactions once the concentrations exceed the critical aggregation concentration. Depending on their design, polymers containing two temperature-sensitive segments covalently linked at each end of the chain can exhibit dual temperature-responsive behavior as a result of conformational changes. Such dual responsiveness can be engineered by combining segments with LCST and UCST characteristics—for example, LCST–LCST, UCST–UCST, or LCST–UCST arrangements, with the latter tailored according to whether the LCST is higher or lower than the UCST. Polymers exhibiting multi-temperature responsiveness with three, four, or even more temperature-responsive characteristics have also been documented. Such polymers are capable of undergoing highly intricate, programmed structural transformations in aqueous solutions. In recent years, these multi-temperature-responsive properties have also been incorporated into other polymeric systems, including gels and nanoparticles [167].

#### 3.2.2. pH-Responsive Polymers

pH-sensitive polymers are a type of intelligent material that experiences significant physical and chemical transformations in response to minor changes in pH levels. These polymers comprise ionizable functional groups that can either take up or release protons, leading to reversible shifts between their charged and neutral forms [168]. pH serves as one of the most frequently utilized stimuli for drug delivery, targeting specific organs (such as the vagina or gastrointestinal tract) or organelles (including lysosomes, Golgi apparatus, and endosomes). It has also been employed to release drug components in response to changed pathological states, such as cancer, inflammation, or ischemia, which are associated with significant shifts in pH levels [169]. In cancer treatment, this characteristic is particularly advantageous because the extracellular environment of tumors is more acidic compared to normal tissues and blood. This acidity results from the hallmark of cancer cells, which is rapid tumor proliferation driven by glycolysis, allowing for the regulated release of drugs [170]. Typically, the pH of tumors falls between 5.8 and 7.8 (with an average of approximately 6.0), while intracellular compartments have even lower pH values (lysosomes: 4.0–5.0; endosomes: 5.0–6.0). As a result, pH-responsive polymeric micelles (PMs) can enhance cellular uptake, boost anticancer effectiveness, and allow for controlled drug release through pH-triggered structural changes within the acidic environment of tumors [171].

The most frequently encountered pH-sensitive structures consist of: (i) chemical bonds that tend to remain quite stable in neutral or alkaline environments but become unstable and susceptible to hydrolysis or cleavage when exposed to acidic conditions. For instance, stimuli-responsive polymer dots can be modified with boronic acid and catechol groups to establish a pH-responsive mechanism, enhanced by reversible diol–diol crosslinking at different pH levels. The pH-sensitive boronate esters display exceptional responsiveness to variations in acidity, rendering them suitable for customized interactions with the acidic tumor microenvironment, thereby facilitating targeted sensing [172]; (ii) polymers that modify their charge characteristics with variations in pH; and (iii) specialized pH-responsive polymers possessing distinctive structural attributes. As these materials change their structure or properties in response to the surrounding pH, the nanocarriers that include them also experience rearrangement, expansion, or breakdown. This mechanism aids in controlled drug release, the removal of protective layers, and disintegration near target locations, ultimately improving drug delivery effectiveness and therapeutic results [173].

Polymeric systems that demonstrate swelling dependent on pH show increased swelling in a simulated fluid with a pH of approximately 7.4, while they exhibit minimal swelling in a pH 1.2 environment. These types of polymeric nanomaterials play a crucial role in creating a gastroretentive drug delivery system designed to allow controlled drug release, reduce gastric side effects, and lower the frequency of administration [174].

Natural PRPs can be categorized based on their source, distinguishing them from synthetic variants. A notable characteristic of many biopolymers is their ability to undergo pH-responsive conformational changes, which often lead to variations in solubility. Natural polymers are often preferred because of their abundance, biodegradability, non-toxicity, biocompatibility, and ease of chemical modification. Representative natural PRPs include hyaluronic acid, alginic acid, heparin, and cellulose derivatives such as carboxymethylcellulose and carboxymethyldextran [175]. Natural polymers are highly regarded for their availability, ability to decompose naturally, compatibility with biological systems, and potential for modification. At the same time, synthetic polypeptide derivatives (PRPs) have been created and are applied in various fields. One notable example is poly(L-glutamic acid) (PGA) [176] and poly(aspartic acid) (PASA) represent biocompatible and degradable pH-sensitive polymers [177,178]. These intelligent polymers have been effectively utilized to create smart active packaging systems that alter the microenvironment for food storage. For example, during fruit storage, acidic compounds such as oxalic acid and carbonic acid may form, causing a decrease in pH. In addition, spoilage-related microorganisms produce acidic metabolites that further lower the pH. pH-sensitive polymers can be designed to respond to these changes by releasing active substances in a controlled manner when needed [177].

#### 3.2.3. Redox-Sensitive Systems

The fundamental concept behind redox-responsive polymeric systems is to exploit the unique variations in redox potentials found between tumor tissues and healthy tissues [179]. Cellular redox homeostasis pertains to the continual balance between oxidizing and reducing agents in cells, which are essential for survival, growth, differentiation, and aging. An excess of reactive oxygen species (ROS) and reactive nitrogen species (RNS) induces oxidative stress, a condition associated with aging and the onset of diseases such as neurodegeneration, cardiovascular disorders, and cancer [180]. Cancer cells exhibit markedly higher levels of reactive oxygen species (ROS) than normal cells. While these elevated ROS contribute to tumor progression, supporting initiation, angiogenesis, and metastasis. Excessive ROS levels beyond a critical threshold can also trigger cytotoxic effects. ROS are mainly produced in the mitochondria via the electron transport chain (primarily Complexes I and III), NADPH oxidases (NOX family), peroxisomes, and enzymes related to the endoplasmic reticulum. The types of ROS produced encompass both free radicals (such as O^2−^, •OH, RO•, ROO•) and nonradicals (like H_2_O_2_, ^1^O_2_, O_3_, ROOH, HOCl, HOBr). Normally, cells maintain redox homeostasis through antioxidant mechanisms such as catalase, superoxide dismutase (SOD), glutathione peroxidase (GSH-Px), and the reduced glutathione (GSH)/oxidized glutathione (GSSG) system. Conversely, in tumors, this balance is frequently disturbed, with increased levels of ROS met with heightened antioxidant responses, especially GSH [180].

The altered redox state leads to significant therapeutic implications. While excessive production of reactive oxygen species (ROS) contributes to oxidative damage to DNA, activation of oncogenes, and disruption of DNA repair processes, thereby promoting cancer development, the imbalance in redox status also makes tumor cells susceptible to increased ROS levels or depletion of GSH, creating a therapeutic opportunity for targeted treatments. Redox-sensitive nanocarriers are developed to take advantage of the heightened intracellular concentrations of GSH, allowing for the controlled release of drugs. This approach raises the levels of drugs within the cells post-treatment, thus improving therapeutic effectiveness while reducing the systemic side effects associated with the original medications [181].

Many redox-sensitive nanocarriers utilize disulfide or diselenide bonds as cleavable connections. Of these, disulfide bonds are the most commonly utilized because of their biocompatibility and reduced toxicity in comparison to selenium-containing groups. Disulfide linkages are frequently incorporated into linker molecules that covalently attach therapeutic agents to the components of the nanocarrier [182]. When high levels of intracellular GSH are present, a thiol–disulfide exchange reaction occurs, causing bond rupture, disassembly of particles, and release of the drug. Although this reaction is thermodynamically favorable (ΔG < 0), it proceeds more slowly than thiol–diselenide exchange, indicating that careful management of disulfide exchange kinetics could enhance drug delivery and improve therapeutic results. In a proof-of-concept study, Dabas and Kanaly (2024) demonstrated that monomers containing disulfide groups, which were specifically designed for the production of nanogels, facilitated the controlled release of the antioxidative enzyme paraoxonase-1 under physiological GSH conditions [182]. Additionally, they maintained the enzymatic activity of the protein payload in stimulated RAW 264.7 macrophages, highlighting that cationic polymeric materials linked by disulfide bonds can act as effective redox-responsive carriers for protein-based therapies, achieving a balance between colloidal stability, encapsulation efficiency, and the preservation of bioactivity [182].

A significant drawback of these systems is their inability to distinguish between cancerous and healthy cells, as both types contain reductive cytosols with millimolar concentrations of GSH. To tackle this issue, the use of tumor-targeting ligands in surface engineering has been suggested to enhance selectivity and reduce off-target repercussions. For instance, angiopep2-aPD-L1@PTX nano-micelles (A2-APM) were recently created by crosslinking anti-PD-L1 antibodies (aPD-L1) and attaching paclitaxel (PTX) to PEG-PLL. The addition of angiopep-2 peptides improved penetration through the blood–tumor barrier, while the reductive microenvironment of GBM triggered the cleavage of the crosslinker and the selective release of aPD-L1 without damaging its structure. At the same time, the dissociation of micelles sped up the release of PTX, which not only caused direct cytotoxic effects but also promoted immunogenic cell death, making tumors more responsive to PD-1/PD-L1 blockade [183].

Cationic polymers, a type of polymeric vector, have garnered considerable interest due to their biodegradability, biocompatibility, and ease of modification when compared to conventional liposomes. By incorporating ROS/GSH-cleavable linkages, redox-responsive polymer nanoparticles (NPs) create a flexible platform for the delivery of intracellular nucleic acids, improving the precision of therapeutics while reducing off-target effects. According to earlier studies, a research team developed a superior polymer called Cys8E, noted for its favorable biocompatibility and responsive nature to GSH, to transport the THZ1 (a covalent inhibitor specific to CDK7). This nanoparticle drug conjugate significantly enhanced THZ1 aggregation and its antitumor efficacy against prostate cancer. In addition, the nanoparticle demonstrated high drug-loading efficiency, excellent stability, and remarkable release capability [184].

Redox-responsive polymers can be utilized to create wound-dressing materials that possess antimicrobial properties and promote wound healing. These materials include redox-responsive degradable hydrogels that are designed by incorporating stimuli-responsive components into the backbone of gel-forming macromonomers. Such hydrogels undergo degradation in response to elevated levels of glutathione (GSH) or reactive oxygen species (ROS), which are typically present at wound sites, in inflamed tissues, or in regions affected by bacterial infections and biofilms. In these environments, the redox imbalance can simultaneously trigger hydrogel degradation and the controlled release of therapeutic agents. Recently, various redox-responsive hydrogels acting as disulfide bond reservoirs have been developed to enhance wound healing, particularly for the topical delivery of therapeutic agents, especially proteins. The inclusion of disulfide bonds in the hydrogel framework offers several advantages: (i) eventual degradation and removal of the hydrogel without inflicting secondary harm to the wound, (ii) provision of dynamic bonds that allow for self-healing, and (iii) regulation of ROS levels in the wound to mediate redox potential and achieve improved wound healing effectiveness. In one study, a redox-degradable hydrogel loaded with the antibacterial peptide vancomycin was synthesized through a straightforward Gram-scale process. The hydrogel structure was based on hyperbranched polyglycerol containing disulfide bonds (SS-hPG), cross-linked with 4-arm polyethylene glycol-thiol (4-arm PEG-SH). Both in vitro and in vivo evaluations confirmed that the vancomycin-loaded hydrogel functioned as an effective antibacterial barrier for wound dressings and significantly accelerated the healing of infected wounds in a mouse model [185].

Despite promising results in preclinical studies, the clinical application of redox-responsive nanomedicines is still limited. While several candidates have entered clinical trials, obtaining regulatory approval remains rare. Two primary obstacles impede translation: (i) their in vivo effectiveness often shows little significant improvement compared to current formulations, and (ii) their intricate design makes large-scale production and quality assurance challenging. Consequently, future research should aim to optimize and streamline nanocarrier design, ensuring reproducibility without sacrificing therapeutic effectiveness, to facilitate broader clinical implementation of stimuli-responsive nanomedicines [186].

### 3.3. Bio-Based and Green Synthesis

#### 3.3.1. Renewable Sources

The concept of biopolymers has developed and now includes materials produced by the polymerization of natural and renewable resources, the polymerization of monomers derived from these resources, or the direct utilization of sustainable macromolecules and their derivatives. These materials come from living organisms like plants, animals, and microorganisms, and they can be acquired either straight from cells or through the chemical synthesis of polymers using bio-based monomers. Biopolymers sourced from plants, such as cellulose, lignin, starch, and hemicellulose, are plentiful and adaptable [187]. These polymers are sourced from a variety of plants, including wheat, rice, and potatoes, among others. For instance, lignin and cellulose are primarily obtained from the agro-industrial waste of lignocellulosic sources. The lignin component of this biomass has been investigated as a starting material for biopolymers such as polyhydroxyalkanoates, polyesters, polyurethanes, and more. On the carbohydrate side, cellulose serves as a significant renewable resource for functional materials. As a homopolymer composed of glucose connected by β (1→4) glycosidic bonds, cellulose exhibits considerable resistance, crystallinity, and mechanical strength. Consequently, much of the research focuses on developing more sustainable extraction methods, followed by physicochemical alterations and modifications, including nano-structuring, to create new functional materials like hydrogels, films, membranes, and coatings [188]. Thanks to their features like biodegradability, biocompatibility, and the ability to customize functionalities, plant-derived biopolymers are gaining popularity in various sectors such as packaging, agriculture, personal care, and biomedical uses.

Microbial pathways produce polymers that naturally biodegrade and have adjustable characteristics, such as polyhydroxyalkanoates (PHAs, with polyhydroxybutyrate, PHB, as a prominent example), polylactic acid (PLA), and bacterial cellulose. These biopolymers are increasingly used in various sectors, including food packaging, medical applications, cosmetics, agriculture, wastewater management, and industrial processes. For example, microalgae are promising raw materials for bioplastic production due to their rapid growth to substantial biomass, lack of direct competition with food crops, and ability to flourish in non-arable conditions like wastewater. They absorb inorganic nutrients to generate proteins, carbohydrates, and lipids, which can be transformed into polymer precursors or extracted as algal polysaccharides for use in bioplastics such as alginate, carrageenan, and agar. Microalgae present an eco-friendly alternative for the commercial production of biopolymers, either through controlled cultivation or harvesting from natural ecosystems. The conversion of algal biomass into bioplastics typically involves several steps, including fermentation, plasticization, blending, and compatibilization.

Microalgae-derived plastics, in particular, are regarded as cost-effective, recyclable, biocompatible, biodegradable, energy-efficient, and flexible. They also offer a reduced carbon footprint and produce minimal toxic by-products, thereby supporting the transition toward a more circular economy. Nevertheless, many existing bioplastics tend to be brittle, exhibiting low melt strength and inadequate barrier properties. Polylactic acid is commonly utilized in the automotive industry because of its mechanical strength [189].

A diverse range of microorganisms—including bacteria, fungi, and yeast—can be engineered to synthesize polymers from renewable carbon sources. A prominent example is polyhydroxyalkanoates (PHAs), biodegradable polyesters produced through microbial fermentation of renewable feedstocks such as sugars and lipids [190].

#### 3.3.2. Green Solvents and Catalysis

In order to prepare standard polymers, a significant amount of fossil fuels, including natural gas and oil, is typically needed as raw materials. This not only strains the finite supply of fossil energy but also increases resource consumption. Furthermore, a significant amount of waste materials is produced during the production and use of traditional polymers, and these wastes are frequently not adequately handled or recycled, leading to significant pollution and adverse environmental effects. Thus, a sustainable and environmentally friendly method of producing biobased polymers is required [189]. The 12 principles of green chemistry were first introduced by Paul Anastas and John Warner in 1998. One of these principles emphasizes minimizing the use of hazardous solvents in chemical processes and preventing the generation of solvent-related waste [185,191].

Growing environmental concerns and the need to create sustainable and green products as alternatives to fossil fuels have led to an increase in the synthesis of bio-based polymers and chemicals in recent years. Making bio-based polymers minimizes reliance on non-renewable feedstocks and maximizes the use of renewable resources. Under the right biological or composting conditions, many bio-based polymers can be made to break down into harmless compounds, reducing pollution and environmental persistence. Sustainability is further improved by designing and creating closed-loop polymers from bio-based materials, which allow for chemical recycling or depolymerization back to monomers. Research on closed-loop, recyclable polymers made from bio-based ingredients has increased recently.

On the other hand, widespread usage of edible biomass feedstocks, including vegetable oils, might put food sources in competition and cause issues with food security. Prioritizing non-food feedstocks and residues, such as lignocellulosic biomass, waste oils and fats, algae, or specialized non-food crops, is one mitigation strategy. To prevent burden shifting, benefits are validated by life-cycle assessment [192]. Utilization of these resources can be greatly improved by employing them as renewable feedstocks, applying targeted chemical modifications, and introducing degradable or dynamically cross-linkable functional groups. These strategies transform them into high-value functional materials and chemicals for research and industrial applications. For example, a study explored non-isocyanate polyurethane (NIPU) precursors derived from non-food aromatics and non-edible oils, Cardanol. This cashew nut shell-liquid derivative combines a flexible C15 aliphatic chain, a rigid aromatic ring, and useful functional groups such as a phenolic hydroxyl and unsaturation. These features enable higher glass transition temperatures and improved thermal stability while maintaining toughness, and cardanol has been widely explored in epoxy, phenolic, benzoxazine, and polyurethane systems [193].

Biopolymers such as polylactic acid (PLA) are inherently flammable and therefore require flame retardants to improve their fire safety. Intumescent flame retardants (IFRs), which typically consist of acid, gas, and carbon sources, are well known for providing effective thermal insulation and smoke suppression. Its flame-retardant effect arises from the formation of an expanded carbonaceous layer during combustion, which further acts as a physical barrier, reducing heat transfer and limiting oxygen penetration, thereby inhibiting the burning of the underlying polymer. From a sustainability perspective, bio-based flame retardants derived from renewable biomass can maintain PLA’s environmentally friendly characteristics and also improve its flame resistance. In one study, a green strategy was developed to enhance both the flame retardancy and toughness of PLA by employing a pH-induced gelling process in water. This approach enabled the deposition of natural latex onto a bio-based core–shell flame retardant (CSFR), yielding a bio-based flame-retardant/natural rubber inorganic–organic hybrid (CSFR-NR) [194].

#### 3.3.3. Eco-Friendly Processing Techniques

Aliphatic polyesters such as poly(butylene succinate) (PBS) and poly(lactic acid) (PLA) are often limited by brittleness, arising from their high crystallinity and susceptibility to thermal degradation. These drawbacks restrict their broader application. Traditional modification strategies frequently employ solution casting with solvents such as chloroform, which raises sustainability concerns [195]. A more sustainable alternative is the incorporation of aromatic units into the polymer backbone, which enhances chain rigidity and thereby improves thermal stability and mechanical performance. This can be accomplished through solvent-free techniques like extrusion, an environmentally friendly and scalable process compatible with high-throughput manufacturing [196].

Solvent-free synthesis methods have also been successfully employed to produce biostable and cytocompatible shape memory polymers, particularly segmented thermoplastic polyurethanes (STPUs) with adjustable thermomechanical properties [197]. STPU synthesis typically involves three key components: small-molecule chain extenders (hard segments), long-chain hydroxyl-terminated macromonomers (soft segments), and coupling agents. The macromonomers provide flexibility, while the chain extenders, upon reaction with coupling agents, form rigid domains stabilized by hydrogen bonding. In solvent-free approaches, STPUs can be synthesized using hexamethylene diisocyanate (HDI), polypropylene glycol (PPG), and triethylene glycol (TEG), eliminating the need for organic solvents such as tetrahydrofuran. This strategy is both straightforward and user-friendly, as it avoids additional titration and strict stoichiometric balancing steps. Moreover, the one-pot reaction setup minimizes preparation time compared with conventional solvent-based methods, offering a more efficient and sustainable pathway to advanced polyurethanes [197].

Despite the benefits of solvent-free synthesis, thermal control remains a key challenge, as managing the heat released during polymerization is difficult, particularly for temperature-sensitive monomers and polymers. This highlights the need to enhance the processability of biobased polymers by broadening their processing window to avoid thermal degradation. A recent study addressed this issue by preparing blends of atactic poly(3-hydroxybutyrate) (a-P3HB) with isotactic poly(3-hydroxybutyrate) (i-P3HB) and poly(3-hydroxybutyrate-co-4-hydroxybutyrate) (P34HB) through solvent-free extrusion. These blends demonstrated an extended processing window, with processing temperatures reduced to 150–160 °C—well below the decomposition onset of i-P3HB—thereby preventing thermal degradation. Moreover, their crystallinity could be tuned between 17% and 70% by adjusting polymer ratios, enabling the fabrication of materials with customizable mechanical properties and elongation at break values of up to 600%. Overall, these results underscore the promise of such blends as sustainable alternatives to conventional plastics, with compatibility across diverse processing techniques including injection molding, extrusion, and fiber spinning [196].

Another eco-friendly strategy for polymer synthesis is enzymatic or organocatalyzed solution polymerization. Enzyme-catalyzed polymerization employs biocatalysts such as lipases, peroxidases, and laccases, while organocatalysis often relies on catalysts such as N, N-dimethyl-4-aminopyridine (DMAP) and N, N′-diisopropylcarbodiimide (DIC). Both approaches operate under mild conditions, typically at ambient temperature and pressure, offering advantages in environmental compatibility and reduced energy consumption. For instance, immobilized Candida antarctica lipase B (iCALB) has been successfully applied as a biocatalyst in the synthesis of 2,5-bis(hydroxymethyl)furan (BHMF), a sustainable rigid furanic compound structurally similar to 2,5-furandicarboxylic acid (FDCA). BHMF serves as a promising biobased building block for the preparation of aliphatic–aromatic polymers, broadening the toolkit of renewable monomers available for sustainable polymer development [198]. However, in this study, only a limited molecular weight was achieved (2100–3100 g mol^−1^).

In a separate study, the copolymerization of BHMF and FDCA, catalyzed either by iCALB or by the combination of DMAP and DIC, resulted in polymers with slightly higher molecular weights [199]. In a more recent study, BHMF was polymerized in bulk for the first time using either the enzyme iCALB or the commercially available catalyst dibutyltin (IV) oxide (DBTO). The resulting BHMF-based polyesters, produced via this solvent-free and sustainable approach, were biodegradable, and their thermal and rheological properties could be tuned by adjusting the number of methylene groups in the aliphatic segment. Despite these advances, challenges remain in maintaining enzyme activity and stability during large-scale production, which continues to hinder broader industrial adoption [195]. Moreover, the high cost of enzyme production and purification further constrains the economic viability of this method for large-scale applications.

Other eco-friendly approaches for the synthesis of polymers include Photopolymerization, Atom Ring-Opening Polymerization (ROP), Transfer Radical Polymerization (ATRP), and click chemistry, which have been reviewed in the literature [190].

## 4. Applications in Key Sectors

**Figure 3:**
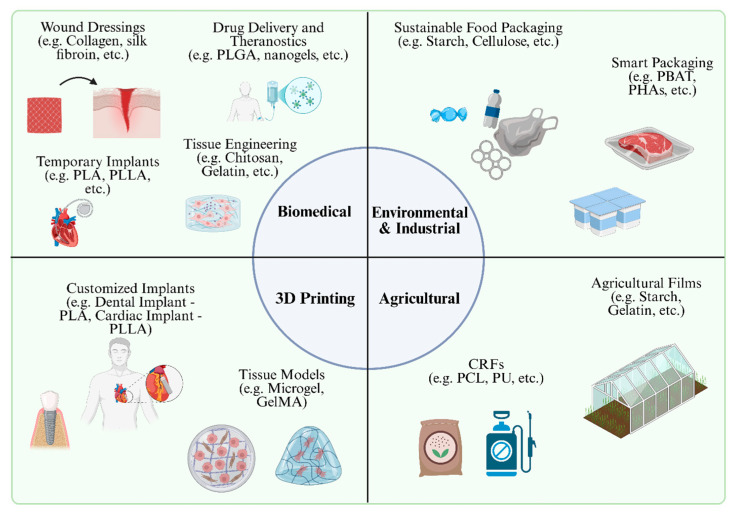
Application of biodegradable and biocompatible polymers in key sectors. Created with Biorender.com.

Due to their versatility in biocompatibility and biodegradability, the development and use of biopolymers have been expanding to multiple domains where performance, safety, and sustainability are critical. The tunable physicochemical properties and advancement in functionalization strategies have enabled biopolymers to be integrated into biomedical applications, their primary application, and other key sectors, such as environmental, industrial, agricultural, and manufacturing additive applications (Figure 3 and Table 3). This section provides an overview of the most prominent applications, particularly biomedical, sustainability-oriented uses for the environment, and industries, and additive manufacturing technologies.

### 4.1. Biomedical Applications

#### 4.1.1. Drug Delivery Vehicles and Diagnostics

Biodegradable and biocompatible polymers have become central to the development of advanced drug delivery and diagnostic systems. Their ability to degrade into nontoxic byproducts, combined with highly tunable physicochemical properties and modifiable surfaces, provides opportunities for sustained release, site-specific targeting, and multifunctional applications. These characteristics position biopolymers as essential materials in the design of controlled delivery vehicles and emerging theranostic platforms [200,201].

Drug release from polymeric systems is governed by polymer erosion, hydrolytic degradation, and diffusion of therapeutic agents through the carrier matrix. More recent innovations have focused on stimuli-responsive materials that respond to pH, redox potential, temperature, enzymatic activity, or light to trigger site-specific release. Such strategies exploit pathological microenvironments, particularly in cancer, to improve drug bioavailability at diseased sites while minimizing systemic exposure and toxicity [202,203].

Among available materials, poly(lactic-co-glycolic acid) (PLGA) remains the most widely used biodegradable polymer due to its FDA approval, tunable degradation rates, and compatibility with diverse therapeutic agents, ranging from small molecules to nucleic acids [204,205,206,207]. Alongside PLGA, smart nanogels represent a new generation of carriers capable of combining the hydrophilicity and flexibility of hydrogels with nanoparticle targeting and release properties. These nanogels can respond dynamically to internal or external stimuli and have shown particular promise in both cancer drug delivery and diagnostic imaging [202].

Surface functionalization strategies, including PEGylation, ligand conjugation, and incorporation of responsive groups, further enhance circulation time, tissue penetration, and cellular uptake [201]. Building on these approaches, recent studies emphasize how chemo-physical tuning of polymer architecture and surface chemistry dictates the biological fate of nanocarriers in vivo. Subtle adjustments to polymer rigidity, hydrophobic-hydrophilic balance, and segmental density can minimize protein adsorption and improve endothelial transport, thereby enhancing pharmacokinetics and biodistribution [208]. In parallel, stealth and pseudo-stealth coatings, including hydrophilic brushes, zwitterionic polymers, and biomimetic coronas derived from albumin or cellular membranes, suppress opsonization and clearance by the reticuloendothelial system [209]. This prolongs systemic circulation and promotes selective accumulation of therapeutic payloads at pathological sites.

At the molecular level, polymer functionalization with stimuli-responsive linkers enables drug release triggered by local pH, enzyme activity, or redox gradients, providing spatially controlled delivery in tumor or inflammatory microenvironments. Meanwhile, optimization of amphiphilic block-copolymer composition and surface charge enhances colloidal stability and interstitial transport, while specific ligand or antibody conjugation promotes receptor-mediated uptake and cellular internalization. Collectively, these molecular-design and surface-engineering strategies integrate polymer chemistry with biological performance to achieve long circulation, predictable biodistribution, active targeting, and controlled release, maximizing therapeutic efficacy and minimizing off-target toxicity [208,209].

The clinical applications of biopolymer-based systems are broad and span oncology, ophthalmology, respiratory medicine, and neurology. In cancer therapy, polymeric nanoparticles, micelles, dendrimers, and drug conjugates have been extensively investigated for both passive accumulation in tumors via the enhanced permeability and retention effect and active targeting through the attachment of ligands such as antibodies, peptides, or folic acid. Multifunctional designs have enabled the co-delivery of cytotoxic drugs with imaging probes, bridging therapy with real-time monitoring and paving the way for personalized theranostics [210,211,212]. In ocular drug delivery, biodegradable microspheres and implants have addressed challenges of rapid clearance from the eye, maintaining therapeutic concentrations in both anterior and posterior segments without repeated invasive procedures [200]. In pulmonary drug delivery, inhalable nanoparticles based on PLGA provide controlled release while protecting drugs from enzymatic degradation, whereas in neurological applications, biopolymer nanoparticles engineered with surface modifications can cross the blood–brain barrier to deliver drugs for neurodegenerative disorders [213,214,215,216,217].

Biopolymer systems, such as multifunctional nanoparticles, have received increasing attention due to their performance, which integrates therapeutic payloads with imaging agents such as fluorescent probes or quantum dots, allowing for simultaneous treatment and disease monitoring. This integration supports early detection, image-guided therapy, and real-time assessment of treatment outcomes, which are critical in advancing precision medicine [202,212].

#### 4.1.2. Tissue Engineering Scaffolds

Biodegradable and biocompatible polymers are pivotal in scaffold-based tissue engineering, where they serve as temporary extracellular matrix (ECM) analogues to support cell adhesion, proliferation, differentiation, and matrix deposition until natural tissue regenerates. These polymers can be natural (e.g., collagen, gelatin, hyaluronic acid, chitosan, alginate) or synthetic (e.g., polylactic acid [PLA], polyglycolic acid [PGA], polycaprolactone [PCL], polylactic-co-glycolic acid [PLGA], polyurethanes, polyphosphazenes). Natural polymers provide intrinsic bioactivity and mimicry of ECM, while synthetic polymers offer tunable degradation, reproducibility, and mechanical strength [218,219,220,221,222]. Increasingly, hybrid and nanocomposite scaffolds combine these advantages, while advanced fabrication methods such as electrospinning, 3D printing, and phase separation yield tailored architectures [223,224,225].

- Bone Tissue Engineering

Bone tissue engineering requires scaffolds with high compressive strength, osteoconductivity, and controlled biodegradation. Synthetic aliphatic polyesters such as PLLA [226,227], PLA, PGA, PLGA, and PCL are widely applied [228,229,230] but often combined with ceramics such as hydroxyapatite (HA) or β-tricalcium phosphate (β-TCP) to mimic bone’s mineralized ECM and to buffer acidic degradation by-products [231,232]. Advanced fabrication strategies, including additive manufacturing and electrospinning, enable scaffolds with interconnected porosity that support vascularization [233,234]. Poly(glycerol sebacate) (PGS) has also been explored for hard-tissue applications through composite formation, where PGS-Bioglass and PGS-HA scaffolds exhibit improved osteoconductivity, enhanced mechanical stiffness (≈0.5 MPa), and support osteoblast proliferation and matrix mineralization while maintaining biodegradability [113]. Nanocomposites, such as PVA, alginate scaffolds reinforced with TiO_2_ nanoparticles, further demonstrate potential for osteogenic induction, antimicrobial activity, and fibroblast viability [235]. Smart scaffolds incorporating bioactive molecules or controlled drug release systems provide multifunctionality for bone regeneration and infection control [236].

- Cartilage Tissue Engineering

Cartilage scaffolds must reproduce viscoelastic properties, nutrient permeability, and the proteoglycan-rich ECM environment [237]. Hydrogels derived from natural polymers such as collagen, agarose, and hyaluronic acid promote chondrocyte phenotype maintenance [238], while PLGA and PCL improve mechanical stability [239]. PGA–hyaluronan scaffolds seeded with mesenchymal stem cells have shown significant chondrogenic stimulation in vivo, making them promising for cartilage repair [240]. The elastomeric and hydrophilic nature of poly(glycerol sebacate) (PGS) facilitates chondrocyte adhesion and extracellular matrix deposition, indicating its suitability for cartilage repair, particularly when blended with natural polymers such as chitosan or collagen. Its moderate degradation rate (≈8–12 weeks) corresponds well with cartilage-regeneration timelines [113]. Advanced strategies, such as electrospun fibers with zonal orientation, replicate the anisotropy of native cartilage, and peptide-functionalized scaffolds enhance glycosaminoglycan and collagen type II synthesis [241,242].

- Neural Tissue Engineering

Neural scaffolds must be soft, elastic, and conductive to guide axonal growth and restore connectivity [243]. PCL and PLGA scaffolds functionalized with conductive polymers, such as polypyrrole, enhance neurite outgrowth and neurocompatibility [244,245]. PGS-based nerve-guidance conduits have shown effective support for Schwann-cell migration and axonal elongation due to their low modulus and hydroxyl-rich surface that favors neural adhesion. Their microporous architecture improves nutrient diffusion and waste removal, reducing inflammation and enhancing axonal regeneration [113]. Hydrogels, due to their hydrated and ECM-like microenvironment, further support neurite extension and synapse formation [246]. Smart biomaterials, including shape-memory and electroactive scaffolds, are being developed to provide electrical stimulation and dynamic cues for neural regeneration [247]. Chitosan derivatives, such as chitooligosaccharides, have also been shown to enhance Schwann cell activity and facilitate nerve regeneration [248,249].

- Skin and Wound Healing

Skin scaffolds are designed to accelerate wound closure, angiogenesis, and re-epithelialization. Natural polymers such as collagen, chitosan, and alginate provide bioactivity and hemostatic properties [250], while PLA and PCL nanofibers fabricated via electrospinning enhance tensile strength and surface area for fibroblast adhesion [251,252]. Hydrogel dressings composed of gelatin, hyaluronic acid, or alginate maintain a moist healing environment and can be engineered for controlled drug delivery [253]. Smart hydrogel scaffolds responsive to environmental cues such as pH and temperature allow on-demand antimicrobial release [254].

In addition to chitosan- and collagen-based systems, glycerol-derived elastomers such as poly(glycerol sebacate) (PGS) and poly(glycerol succinate) (PGSuc) provide biocompatible and flexible platforms for wound dressings and soft-tissue regeneration. Their hydroxyl-functional surfaces enhance fibroblast adhesion and angiogenesis, while their controlled degradation maintains mechanical integrity during the healing process [113]. PGS-based wound dressings and adhesives also exhibit conformal contact with irregular wound geometries and strong wet adhesion inspired by gecko-like mechanics, making them suitable for hemostatic or post-surgical applications. Such PGS membranes act as temporary epidermal barriers, preventing infection and dehydration while promoting re-epithelialization [113].

- Vascular and Cardiac Tissue Engineering

Vascular scaffolds require compliance and anisotropy to prevent thrombosis and intimal hyperplasia [255]. Electrospun fibrous tubes of PLLA support endothelialization, while anticoagulant-functionalized lumens enhance hemocompatibility [256]. In cardiac tissue engineering, scaffolds must withstand cyclic strain and fatigue while supporting synchronized cardiomyocyte contraction [257]. Conductive elastomers and hydrogels promote electrical coupling and pacing [258,259]. Shape-memory polymers are also explored for minimally invasive vascular stents and cardiac patches that expand in situ [260].

Beyond conventional polyesters, PGS and PGSA (poly(glycerol sebacate acrylate)) have emerged as benchmark elastomers for cardiac and vascular scaffolds. PGS exhibits mechanical compliance and elasticity comparable to native myocardium (0.05–1.2 MPa modulus, >300% elongation), enabling cyclic deformation without mechanical failure. Porous “accordion-like” honeycomb PGS architectures mimic the anisotropy of the heart wall and support spontaneous, synchronous beating of cultured cardiomyocytes [113]. Its hydrolytic degradation yields nontoxic products, while hydroxyl-rich surfaces promote endothelialization and integration. For small-diameter vascular grafts, PGS’s compliance minimizes stress mismatch, and PGSA’s UV-curable chemistry allows precision micro-patterning and rapid scaffold fabrication for 3D printing and photo-curing processes [113].

- Other Soft Tissue Applications

In bladder and urethral scaffolds, elastomeric polymers such as poly(glycerol sebacate) and polyurethanes replicate compliance and resilience [261,262]. For tendon and ligament repair, aligned fibrous scaffolds made from PLGA and PLLA guide fibroblast orientation and new collagen deposition [263,264]. Corneal scaffolds require transparency and hydration, achieved with hydrogels engineered for refractive clarity and biocompatibility [265].

Glycerol-based aliphatic polyesters, particularly poly(glycerol sebacate) (PGS) and poly(glycerol succinate) (PGSuc), form a distinctive subgroup of biodegradable polyesters used in soft-tissue engineering. Their elastomeric and compliant nature allows close replication of soft-organ mechanics, making them attractive for bladder, urethral, and skin substitutes as well as flexible nerve and ligament supports. These polymers degrade gradually into biocompatible products, minimizing inflammation and eliminating the need for device removal. Their cross-linked structures can be tuned by curing temperature and time to match the stiffness or resilience required for different soft tissues [113]. Beyond musculoskeletal tissues, PGS thin films have been employed in tympanic membrane and retinal tissue reconstruction, where optical transparency and flexibility support epithelial attachment and light transmission, highlighting the polymer’s potential in ophthalmic and auditory repair [113].

- Smart and Stimuli-Responsive Scaffolds

Recent research highlights “smart” scaffolds with shape-memory, electroactivity, and stimuli-responsiveness (pH, temperature, magnetic, or electrical cues). These scaffolds not only act as ECM analogues but also deliver dynamic signals, minimally invasive deployment, and controlled drug release [266,267].

Recent advances have extended glycerol-based polyesters into stimuli-responsive and smart scaffolds. Functionalizing PGS and poly(glycerol succinate) (PGSuc) backbones with photo-crosslinkable or thermoresponsive moieties enables shape-memory behavior, electrical conductivity, and on-demand degradation for next-generation biomedical devices. Related short-chain diol-dicarboxylic acid polyesters, such as poly(glycerol adipate) and poly(glycerol azelate), provide intermediate stiffness and faster resorption rates, bridging the mechanical gap between rigid thermoplastics and hydrogels [113].

In parallel, linear aliphatic polyesters derived from short-chain diols and dicarboxylic acids, such as poly(butylene succinate) (PBS), poly(butylene adipate) (PBA), and poly(ethylene succinate) (PESu) exhibit tunable stiffness, crystallinity, and degradation rates depending on the monomer chain length. These materials hydrolytically degrade into biocompatible diols and acids (e.g., succinic, adipic) and have been employed in resorbable sutures, fixation pins, and slow-release coatings [113]. Their higher crystallinity and mechanical stability (modulus ≈ 200–400 MPa) make them suitable for structural or load-bearing applications, whereas PGS-type triol systems provide complementary elasticity for soft-tissue scaffolds.

#### 4.1.3. Temporary Implants and Wound-Healing Materials

Biodegradable polymers underpin the design of temporary implants and wound healing systems, enabling devices that provide transient structural or biological support before degrading safely in vivo. This reduces the need for secondary removal surgeries, lowers complication risks, and allows controlled synchronization of device degradation with the tissue regeneration timeline [7,268]. The progression from inert to bioactive biomaterials has expanded the potential of polymers to actively participate in healing rather than simply acting as passive supports [269].

- Temporary Implants

In orthopedics, biodegradable polymers are central to temporary fixation devices such as plates, screws, rods, sutures, and stents [270]. Polyesters including PLA, PLLA, PGA, PLGA, and PCL, are the most widely applied, with elastic moduli closer to bone than metals, reducing stress shielding while ensuring gradual transfer of load to regenerating tissue [271,272,273,274]. PLA in particular demonstrates excellent biocompatibility, degrading into lactic acid that is safely metabolized or excreted [275]. Its resorption rate can be tuned Via stereochemistry (L-, D-, DL-forms) and copolymerization (PLGA), aligning degradation kinetics with bone or soft tissue healing [276].

Composites such as PLA-hydroxyapatite (HA) and PLLA-HA demonstrate improved mechanical properties and osteoconductivity, promoting bone integration while maintaining degradability [277,278]. Injectable thermosensitive implants based on mPEG–PLGA copolymers form gels in situ at body temperature, enabling localized and sustained release of proteins or drugs, expanding the role of implants beyond mechanics to controlled therapy [279].

Biodegradable implants such as screws, plates, and pins achieve effective fixation with fewer long-term complications compared to permanent metal devices [280,281]. Short-term host responses resemble those of inert implants, but inflammation resolves as the polymer degrades, unlike metallic systems that may cause ion release and chronic tissue reactions [282]. Rare complications, such as foreign-body responses or osteolysis, are more commonly associated with fast-degrading PGA, whereas PLA-based implants exhibit better long-term tolerance [283].

- Wound-Healing Materials

Polymer-based wound dressings now function as bioactive interfaces with the wound bed. Hydrogels, nanofibrous mats, foams, and films provide moisture retention, exudate absorption, and antimicrobial barriers while promoting cell adhesion, angiogenesis, and re-epithelialization [284,285,286,287]. Natural polymers demonstrate inherent biocompatibility and bioactivity but consistently exhibit poor mechanical properties that necessitate blending with synthetic polymers. ECM with PEUU fibers achieved 80–187 kPa tensile strength, while PVA–hydroxyapatite composites improved mechanical properties by up to 64% [288,289]. Stimulus-responsive and smart hydrogels, which perform self-healing, conductive, or responsive to pH, temperature, and ions, are emerging, and can dynamically adapt to wound microenvironments. Self-healing hydrogels and bioadhesives, including cyanoacrylate blends, provide alternatives to sutures by offering conformal closure and antibacterial activity [290].

### 4.2. Environmental and Industrial Uses

#### 4.2.1. Sustainable Packaging Alternatives

The global packaging sector, dominated by petroleum-derived plastics, is a primary contributor to environmental pollution, greenhouse gas emissions, and the generation of persistent microplastics [291]. Conventional plastics such as polyethylene, polypropylene, and polystyrene provide excellent mechanical and barrier properties but resist degradation for centuries, disrupting terrestrial and aquatic ecosystems [292]. Biodegradable and biocompatible polymers present a sustainable alternative by offering comparable packaging performance while degrading under controlled conditions into harmless by-products such as carbon dioxide, water, and biomass [293].

- Natural Biopolymers for Packaging

Natural polymers, including starch, cellulose, chitosan, alginate, carrageenan, pectin, and proteins such as gelatin and casein, are renewable, biodegradable, and biocompatible. They can be processed into edible films, coatings, and biodegradable packaging materials with inherent bioactivity [294]. Edible films and coatings made from proteins and lipids add functionality by improving sensory quality and acting as carriers for antioxidants and antimicrobials, reducing the need for synthetic additives [295,296,297]. Starch remains the most widely used due to its abundance and low cost, though native starch films are brittle and moisture sensitive. Converting starch into thermoplastic starch (TPS) through plasticization improves flexibility and processability, positioning TPS as a scalable replacement for single-use plastics [298,299]. Cellulose derivatives yield transparent and strong films [300,301], while chitosan-based materials provide intrinsic antimicrobial activity and valorize seafood industry waste streams [302,303]. Alginate continues to expand its role in edible and biodegradable food coatings [304].

- Synthetic Biodegradable Polyesters

Polylactic acid (PLA), polycaprolactone (PCL), and poly(butylene adipate terephthalate) (PBAT) are among the most widely commercialized [305,306,307]. PLA, derived from lactic acid, is the most established, offering mechanical and optical properties similar to polyethylene terephthalate (PET), though limited by brittleness and thermal instability [308]. Blending PLA with PET decreases tear strength and increases impact strength, while increasing color and decreasing transparency [309]. PCL offers flexibility and compatibility with blends, though it is slow in degradation [310,311].

- Blends, Composites, and Compatibilization

The inherent weaknesses of pure biopolymers, such as low mechanical strength, poor thermal stability, and weak barrier properties, are addressed through blending, compatibilization, and composite strategies. PLA–starch and PBAT–TPS blends, when compatibilized, exhibit improved strength and flexibility [312,313]. Lignocellulosic fillers (coffee grounds, nanocellulose, date stones) and nanoclays enhance tensile properties, reduce permeability, and improve thermal stability [314,315,316,317]. Eco-friendly surface modifications with rosin or stearic acid improve filler–matrix compatibility while retaining biodegradability [318,319]. Nanocomposites, particularly those incorporating nanoclays, silica, and graphene derivatives, significantly improve oxygen and water vapor barrier performance, making biopolymer films more competitive with petrochemical plastics [320,321,322].

- Active, Antioxidant, and Smart Packaging

Packaging is increasingly designed to extend shelf life and ensure food safety. Active packaging integrates antimicrobial or antioxidant agents directly into films. Biopolymer matrices embedded with nanoparticles (silver, TiO_2_, ZnO) inhibit microbial growth [323], while natural additives such as essential oils, phenolic compounds, and plant extracts reduce spoilage [324,325,326,327]. PLA films enriched with tocopherol or olive leaf extract exemplify antioxidant-active packaging, controlling oxidative degradation and extending freshness [328,329]. Smart packaging incorporates pH- or temperature-sensitive indicators, conductive hydrogels, and self-healing materials to monitor food quality and freshness in real time [330,331,332].

- Industrial Applications and Cross-Sector Relevance

Food packaging remains the most extensive application area, with biodegradable films already commercialized in modified atmosphere packaging (MAP) systems [333]. In pharmaceuticals, biodegradable polymers such as starch, PLA, and gelatin provide safe, non-toxic, and protective packaging [334]. Industrial applications extend beyond food and medicine to include agricultural films, disposable containers, and eco-friendly paperboard coatings [335,336,337].

Beyond biomedical applications, short-chain diol-dicarboxylic acid polyesters such as poly(butylene succinate) (PBS), poly(ethylene succinate) (PESu), and poly(butylene adipate) (PBA) have attracted attention as renewable and biodegradable thermoplastics. They are synthesized via condensation of bio-derived monomers, such as succinic acid from microbial fermentation and 1,4-butanediol from glucose hydrogenation. These polymers combine thermal processability, mechanical strength, and controlled biodegradation [113].

PBS and PESu exhibit semicrystalline structures and hydrolytic degradation into non-toxic small molecules, making them suitable for biomedical devices and sustainable packaging. Their mechanical moduli (200–400 MPa) exceed those of PGS-type elastomers, enabling their use in rigid containers, resorbable plates, and eco-friendly films. Furthermore, integrating diol-based polyesters into PLA or starch blends enhances flexibility and compostability, bridging the gap between high-performance biomedical polymers and environmentally sustainable materials [113].

#### 4.2.2. Agricultural Applications

Agriculture is a major consumer of plastics and chemical inputs, contributing to plastic pollution, inefficient nutrient use, and soil and water degradation [338]. Biodegradable and biocompatible polymers have emerged as key tools to address these challenges, offering applications ranging from mulch films and crop covers to controlled-release fertilizers and pesticides, superabsorbents, and nanocarriers [339]. These innovations reduce environmental impact, improve input-use efficiency, and align with policy and sustainability targets for climate-smart agriculture [340].

- Biodegradable Mulch Films and Crop Covers

Mulching improves soil temperature regulation, moisture retention, and weed suppression [341,342]. Conventional polyethylene films, however, accumulate as persistent waste in soil [343,344,345]. Biodegradable mulch films based on starch, cellulose, PLA, PHAs, and their blends degrade naturally without leaving toxic residues, making them attractive alternatives [346,347]. Field studies show that these films improve crop yields, water-use efficiency, and soil structure while reducing the need for herbicides [341]. Beyond mulching, biopolymer-based crop covers and greenhouse films provide protection while mitigating microplastic pollution [348,349,350].

- Controlled- and Slow-Release Fertilizers

Over-application of fertilizers leads to nutrient leaching, runoff, and volatilization, causing eutrophication and greenhouse gas emissions [351]. Biodegradable polymers are used as coatings or carriers in controlled-release fertilizers (CRFs), synchronizing nutrient delivery with crop demand [352]. Starch-based hydrogels form three-dimensional networks that absorb water and gradually release nitrogen, phosphorus, and potassium, also acting as soil conditioners [353,354,355]. Coatings made of polyvinyl alcohol (PVA), PLA, gelatin, or gum arabic significantly reduce nutrient losses, enhance microbial biomass, and improve yields in maize and other crops [356]. Electrospun PVA/PLA core–shell nanofibers encapsulating NPK have demonstrated efficient nutrient release and improved growth in lettuce under field conditions [357].

Stimuli-responsive coatings represent a new generation of CRFs. Temperature-sensitive polyurethanes derived from polycaprolactone (PCL) regulate nitrogen release depending on soil conditions [358]. Similarly, bio-based coatings that are responsive to pH, enzymatic activity, or moisture enhance nutrient-use efficiency while minimizing environmental leakage [359,360]. Comparative studies distinguish between slow-release fertilizers (SRFs), which release nutrients gradually, and CRFs, which synchronize release with physiological crop needs, both of which benefit from biodegradable polymer matrices [361,362].

### 4.3. Additive Manufacturing and 3D Printing

Additive manufacturing (AM), commonly referred to as 3D printing, has transformed biomedical engineering by enabling the production of customized, patient-specific implants, scaffolds, and tissue models with unprecedented precision [363]. Unlike traditional subtractive manufacturing, AM constructs objects layer by layer, allowing intricate geometries, controlled porosity, and integration of bioactive agents. Biodegradable and biocompatible polymers, particularly PLA, PCL, and hydrogel-based systems, are central to these advances due to their tunable mechanics, processability, and safe degradation pathways [364,365].

#### 4.3.1. Custom Implants

PLA and PCL dominate as thermoplastic feedstocks in fused deposition modeling (FDM) and selective laser sintering (SLS) [366,367]. PLA offers rigidity, biodegradability, and printability, making it suitable for craniofacial plates, dental implants, and fixation devices, though it degrades relatively quickly [368]. PCL, with slower degradation and higher elasticity, is employed in load-bearing applications, cartilage substitutes, and bone scaffolds, where gradual resorption is advantageous [369,370]. PLA/PCL composites reinforced with bioactive fillers such as hydroxyapatite (HA) or bioglass enhance osteoconductivity and integration, addressing the limitations of pure polymers [371,372]. Custom implants fabricated via AM have shown promising outcomes in orthopedics, including hip, knee, and spinal implants, offering lightweight, porous structures that mimic trabecular bone and improve osteointegration [373,374,375].

#### 4.3.2. Dental and Craniofacial Applications

AM has also advanced dental and oral regeneration. Biodegradable polymers such as PLA, PCL, and PLGA, often combined with hydrogels or ceramics (e.g., nano-HA), are used to produce 3D-printed constructs for periodontal, alveolar bone, and pulp regeneration [376,377,378]. Multi-material strategies allow region-specific repair, such as scaffolds supporting both pulp regeneration and alveolar bone healing, leading to multifunctional constructs with enhanced regenerative efficacy [379].

#### 4.3.3. Tissue Models and Hydrogel Bioinks

Hydrogel-based polymers serve as the backbone of bioprinting tissue models. Materials such as gelatin methacryloyl (GelMA), alginate, hyaluronic acid, collagen, and PEG derivatives replicate extracellular matrix (ECM) properties, providing cell-encapsulating environments for constructing vascularized tissues, cartilage replacements, and organ-on-chip systems [380,381,382,383]. Despite their bioactivity, hydrogels often lack mechanical strength, necessitating hybrid approaches where PLA or PCL frameworks provide structural stability while hydrogels supply biological functionality [384,385,386,387].

#### 4.3.4. Advanced Additive Manufacturing Technologies

Different AM technologies cater to specific biomedical needs. FDM is widely used for thermoplastics like PLA [388], while stereolithography (SLA) and digital light processing (DLP) enable high-resolution printing of photo-crosslinkable hydrogels [389,390]. Selective laser sintering (SLS) produces porous implants with controlled architecture, and binder jetting allows large-scale customization of surgical models [391]. Advances in photoinitiator chemistry have improved the safety and resolution of hydrogel-based printing for tissue engineering [392]. Furthermore, multi-material additive manufacturing (MMAM) enables the simultaneous fabrication of composites, yielding multifunctional implants [393,394,395].

#### 4.3.5. Stimuli-Responsive and 4D Printing

Recent developments in stimuli-responsive polymers (SRPs) have introduced 4D printing, where constructs change properties or geometry over time in response to external triggers such as temperature, pH, or light [396,397,398]. PLA and PCL have been functionalized with shape-memory capabilities [399], while hydrogels provide swelling/shrinking responses, opening opportunities for dynamic tissue scaffolds and smart implants that adapt in vivo [400].

**Table 3:** Applications of polymers in diverse sectors: medicine, agriculture, and environment.

Biopolymer (Family)	Key Property	Typical Forms	Representative Application	References
Medicine				
PLGA, PEG, nanogels, PEGylated carriers	Biocompatible, biodegradable, tunable degradation and surface chemistry, stimuli-responsive	Nanoparticles, micelles, dendrimers, nanogels	Drug delivery and theranostics (cancer, ocular, pulmonary, neurological)	[200,201,202,203,204,205,206,207,208,209,210,211,212,213,214,215]
Collagen, gelatin, hyaluronic acid, chitosan, alginate (natural); PLA, PGA, PCL, PLGA, PU, polyphosphazenes (synthetic)	Support cell adhesion, tunable degradation, and mechanical strength	Hydrogels, electrospun fibers, 3D-printed scaffolds, nanocomposites	Tissue engineering (bone, cartilage, neural, skin, vascular, cardiac)	[216,217,219,220,221,222,223]
PLA, PLLA, PGA, PLGA, PCL, PGS, PGSuc, short-chain diol-dicarboxylic acid polyesters (PBS, PESu, PBA)	Biodegradable and biocompatible; tunable stiffness and elasticity; hydrolytic degradation into non-toxic by-products	Composite scaffolds, fixation plates and screws, nanocomposites, elastomeric films	Temporary implants (orthopedic, dental) and hard/soft-tissue scaffolds (bone, cartilage, cardiac)	[113,224,225,226,227,228,229,230,231,232,233,234,235,236]
PGS, PGSuc, PU, collagen, chitosan, gelatin, alginate, silk fibroin, PVA composites	Elastomeric, bioactive, hemostatic, flexible; tunable cross-linking and hydroxyl-functional surfaces	Hydrogels, foams, nanofibrous mats, bio adhesives, and elastomeric membranes	Wound healing and soft-tissue regeneration (skin, vascular, cardiac, bladder, neural)	[113,237,238,239,240,241,242,243]
Starch, cellulose, chitosan, alginate, carrageenan, gelatin, casein, PESu, PBS blends	Renewable, biodegradable, edible, antimicrobial; tunable crystallinity and processability	Films, coatings, TPS blends, edible composites	Sustainable food packaging (active, antioxidant, smart films)	[113,244,245,246,247,248,249,250,251,252,253,254,255,256,257]
PLA, PCL, PBAT, PHAs, PBS, PESu, and blends	Biodegradable synthetic polyesters with tunable mechanics and barrier properties	Blends, composites, and nanocomposites for thermal and mechanical stability	Packaging (eco-friendly, industrial, agricultural, pharmaceutical)	[7,113,258,259,260,261,262,263,264,265,266,267,268,269,270]
Starch, cellulose, PVA, PLA, chitosan, gelatin, gum Arabic, PU, PCL coatings	Moisture retention, nutrient-controlled release, soil biodegradability	Mulch films, hydrogels, coatings, nanocarriers	Agricultural films, controlled-release fertilizers, pesticide carriers, biosorbents	[272,273,274,275,276,277,278,279,280,281,283,284,285,286,287,288,289,290,291,292,293,294,295,296,338,339,340,341,342,343,344,345,346,347,348,349,350,351,352,353,354,355,356,357,358,359,360,361,362]
PLA, PCL, PLGA, PGS, PGSuc, PBS, GelMA, alginate, collagen, PEG derivatives	Printable, biocompatible, degradable; photo-/thermo-crosslinkable	FDM filaments, hydrogel bioinks, composites for 3D/4D printing	Additive manufacturing (implants, dental, tissue models, 4D printing)	[227,273,297,298,299,300,301,302,303,304,305,306,307,308,309,310,311,312,313,315,316,317,318,319,320,321,322,323,324,325,326,327,328,329,330,331,332]
Environment				
Chitosan	Cationic, chelating, antimicrobial	Beads, powders, membranes	Waste water treatment (dyes, heavy metals), antimicrobial food wraps	[401]
Alginate	Gel-forming, non-toxic, high water absorption	Beads, films, hydrogels	Heavy metal removal, encapsulation of microbes, food packaging	[402,403]
Starch-based blends,	Biodegradable, renewable, adhesive	Films, foams, hydrogels, composites	Controlled-release fertilizers/urea, compostable packaging, bioplastic blends	[404]
Silk fibroin	Amphiphilic, High mechanical strength	Membrane, films, gels, films, composites	Heavy-metal adsorption membranes; water purification	[405]
PHA, PCL	Fully biodegradable, thermoplastic	Films, fibers, molded parts	Biodegradable packaging, soil-biodegradable plastics, mulch films	[406,407]
Industry				
Starch/PVA	Adhesive, high tensile strength, hydrophobicity, flexibility	Pastes, glues	Paper, food packaging adhesives, corrugation	[408,409,410]
PLA	Thermoplastic, compostable, high tensile strength	Films, fibers, molded parts	Packaging, bioplastic, 3D printing filaments	[411,412]
PHA	Versatile thermoplastics	Films, fibers, composites	Bioplastics, single-use items, paper coatings	[413]
Cellulose derivatives	Renewable, water-soluble	Films, fibers, membranes, composites	Packaging, textile finishing, paper coatings	[414,415]
Polyurethane	Adhesive, flexible, high mechanical strength	Foams, composites	Coatings, furniture	[416,417]

## 5. Challenges and Limitations

Polymers are unique and constantly developing materials that have gained significant attention because of their advantageous mechanical and physicochemical properties, such as tensile strength, mechanical modulus, and degradation rate, all of which can be customized for specific applications by varying polymerization conditions [418]. Nonetheless, despite these advantages, a few challenges remain associated with their use, which impose significant constraints.

### 5.1. Mechanical Property Enhancement

One of the most persistent disadvantages of biodegradable polymers is their lower mechanical strength as compared to conventional, petroleum-derived alternatives. To make biodegradable composites feasible for practical applications, studies have stressed the urgent need for enhanced mechanical reinforcement and better management of structure-property interactions [5]. In biomedical settings, such limitations translate into implants or scaffolds that lose strength too quickly, while in packaging they lead to short shelf-life under humid or mechanical stress conditions.

For instance, PLA shows good tensile strength but breaks at under 10% elongation, reflecting its brittle nature. That is why various research have explored things like adding cellulose nanocrystals or plasticizers to boost flexibility with one study reporting improvement in elongation from 6% to 140–190% [419]. Even so, the mechanical properties of PLA do not always match those of fossil-based polymers, and its excessive brittleness can make performance difficult to accurately predict [420]. To mitigate this, a promising approach in recent experiment used the reactive compatibilization of PLA with ductile polymers like PBAT or PCL using epoxy chain extenders (e.g., Joncryl) to chemically bond phases and enhance toughness [421].

It has also been shown that the incorporation of natural fibers into PLA can enhance mechanical strength, but the resulting composites exhibit pronounced sensitivity to hydrothermal aging [422]. In a study analyzing PLA/flax bio composites, specimens immersed at 20 °C, 35 °C, and 50 °C for up to 51 days showed that while low-temperature exposure induced primarily reversible swelling and plasticization effects, higher temperature conditions triggered irreversible molecular degradation, marked by hydrolysis, increased crystallinity, crack formation, and a sharp decline in mechanical integrity. Notably, a 10 wt % fiber addition extended the effective lifespan by approximately 230 percent at 50 °C, although longer exposure ultimately accelerated deterioration [423]. To counteract these effects, experimental work now focuses on fiber surface modifications, such as silane coupling agents, plasma treatments, or hydrophobic sizing to strengthen interfacial adhesion and limit moisture uptake, thereby reducing property loss during aging [424].

A persistent gap remains between laboratory advances and consistent performance under physiological or service conditions. A fundamental issue is mechanical robustness versus degradability: materials engineered to erode or resorb frequently lose load-bearing capacity or barrier function before the application timeline is complete. This trade-off is evident in biomedical contexts (e.g., vascular scaffolds and temporary implants) where stiffness retention and fatigue resistance are as critical as bioresorption kinetics, and in packaging where oxygen/water vapor barriers degrade as crystallinity or morphology evolves during use. Current literature highlights that addressing these challenges will likely require higher crystallinity, strategic blending, and optimized nanofiller loading to achieve a practical balance between strength and biodegradability [425,426].

### 5.2. Degradation Kinetics and By-Products

Another critical limitation is the difficulty of precisely controlling degradation rates. In drug delivery or tissue engineering, premature loss of mechanical integrity can compromise clinical outcomes, while delayed degradation can trigger chronic inflammation or necessitate surgical retrieval. PLGA undergoes autocatalytic hydrolysis, and during this process, ester bond cleavage produces lactic acid and glycolic acid monomers that accumulate within the bulk of the material [427]. These acidic by-products lower the local pH, which in turn accelerates further hydrolysis, creating a self-reinforcing degradation cycle [428].

The biological consequences of these by-products are significant. Accumulation of lactic and glycolic acid has been shown to recruit immune cells, polarize macrophages toward a pro-inflammatory (M1) phenotype, and trigger localized irritation in surrounding tissues [429]. In tissue engineering applications, this can compromise scaffold integration or prolong inflammatory phases of wound healing. Similarly, in drug delivery systems, acidic microenvironments may destabilize sensitive drugs, altering release profiles and efficacy [428]. Recent studies show that incorporating mild basic buffers such as magnesium hydroxide (Mg(OH)_2_) or calcium carbonate within the matrix can neutralize acidic oligomers in situ and markedly reduce local pH drops without compromising polymer integrity [430].

By-product hazards extend beyond biomedical uses to the environmental setting. While polymers such as PLA or PHAs are marketed as biodegradable, their breakdown in real-world conditions frequently yields oligomers, organic acids, or microplastic particles that may survive and interact with ecosystems. [431]. Limited composting infrastructure and changing environmental conditions exacerbate degradation, creating concerns about the ecological safety of these materials [431]. For example, in the case of biodegradable mulch films, typical biodegradability testing procedures have significant drawbacks because they are frequently performed under controlled laboratory conditions and rely on indirect metrics that do not account for soil properties. Field performance is heavily impacted by pH, moisture, salinity, and microbial diversity; hence, laboratory findings may not adequately reflect actual degradation behavior. Even when polymers breakdown in soil, the long-term consequences of remainder matter remain largely unknown, emphasizing the necessity for field-based testing methodologies that mirror actual circumstances and account for any residual impacts [432].

Bio-based polyesters degrade slowly in packaging applications, and structural modification through techniques such as copolymerization or mixing can be a useful way to speed breakdown. These efforts can result in biodegradable polymers under mild conditions, such as home composting or direct soil burial [335].The current study also reveals that introducing pro-oxidants or natural polymers can improve anaerobic and aerobic degradation, whereas pre-treatment with UV radiation, heat, or light accelerates the breakdown. However, a few researchers have described how efficiently such approaches increase the degradation rates of regularly used polymers like PLA and PGA, leaving significant data gaps [433].

These findings highlight the importance of understanding not only degradation kinetics, but also the nature, accumulation, and impact of byproducts in order to advance both biological safety and environmental sustainability.

### 5.3. Scalability and Cost

Even when the performance requirements are reached, scaling up manufacturing remains a major problem. Biodegradable polymers such as PLA, PHA, and PCL are currently more expensive to manufacture than their petroleum-based counterparts, largely due to higher feedstock costs and energy-intensive fermentation or processing requirements [434]. Techno-economic analyses (TEA) indicate that feedstock cost and downstream recovery dominate the cost of biopolyesters. For PHA, raw materials typically account for over 40% of the total production cost [435]. Consequently, scale-up strategies now emphasize the use of low-cost renewable substrates such as agricultural wastes or industrial side-streams, improved microbial productivities, and simplified extraction and purification steps to achieve market competitiveness [435].

Industrial adaptation is further slowed by incompatibility with existing processing lines, meaning costly modifications to equipment are often necessary [5]. For packaging and agriculture, cost competitiveness is paramount; without significant reductions, adoption beyond niche applications will remain limited. For example, in agriculture, biodegradable mulch films have been developed as alternatives to polyethylene; however, their large-scale use remains limited because they are expensive, difficult to manage, and often require specialized equipment for application [432].

Despite reports of rapid growth in the amount of biodegradable plastics that are being produced (about 1.1 million tons in 2022), they are still comparatively expensive and hard to scale to match fossil-derived plastics [5]. Schick et al. outlined how biopolymers are not yet translating into widely available products. It points to economic pressures, ecological concerns, and the need for “degradation by design” approaches, where the manufacturing process and degradation behavior are planned hand in hand [434]. Innovative approaches, such as energy-efficient processing technologies, including out-of-autoclave (OoA) methods, are proposed as viable pathways to reduce environmental impact while supporting circular economy adoption [436].

Nonetheless, despite these promising advances, scaling such techniques remains a significant challenge, underscoring the need for systemic solutions that integrate technical innovation with economic feasibility. Poly(glycerol sebacate) (PGS) serves as a key example in this regard. Recent progress in the scalable synthesis of PGS demonstrates how process optimization, particularly catalyst-assisted and mathematically optimized synthesis methods can substantially reduce production time and energy consumption, offering a promising pathway for industrial-scale polymer manufacturing [437]. Economic feasibility analyses of biodegradable mulch films further emphasize this challenge, indicating that widespread adoption may only be possible with government intervention through subsidies, incentives, or targeted marketing programs to support farmers [432].

### 5.4. Regulatory and Standardization Issues

Regulatory frameworks and standardized testing remain critical yet underdeveloped for both biodegradable and biocompatible polymers. In the environmental sector, several internationally recognized specifications exist, including ASTM D6400 [ASTM D6400 12; Standard Specification for Labeling of Plastics Designed to be Aerobically Composted in Municipal or Industrial Facilities. ASTM International: West Conshohocken, PA, USA, 2012.], which sets criteria for biodegradability based on the requirement that 90% of carbon atoms must be mineralized to CO_2_ within 180 days [438,439]. This standard is supported by a suite of related ASTM protocols, that test biodegradability under aerobic, anaerobic, and composting conditions. They include [439]:

- D5209 [ASTM D5209-92; Standard Test Method for Determining the Aerobic Biodegradation of Plastic Materials in the Presence of Municipal Sewage Sludge (Withdrawn 2004). ASTM International: West Conshohocken, PA, USA, 1996-2025],
- D5338 [ASTM D5338-21; Standard Test Method for Determining Aerobic Biodegradation of Plastic Materials Under Controlled Composting Conditions. ASTM International: West Conshohocken, PA, USA, 2021],
- D6002 [Standard Guide for Assessing the Compostability of Environmentally Degradable Plastics. ASTM International: West Conshohocken, PA, USA, 1996],
- D5988-03 [Standard Test Method for Determining Aerobic Biodegradation of Plastic Materials in Soil of Plastic Materials or Residual Plastic Materials After Composting. ASTM International: West Conshohocken, PA, USA, 2003], and
- D6954 [Standard Guide for Exposing and Testing Plastics That Degrade in the Environment by a Combination of Oxidation and Biodegradation. ASTM International: West Conshohocken, PA, USA, 2018].
  Comparable frameworks have been developed by the Bureau of Indian Standards (BIS) and the International Organization for Standardization (ISO), with specifications adopted across countries to harmonize evaluation methods [438]:
- ISO 14851 [Determination of the Ultimate Aerobic Biodegradability of Plastic Materials in an Aqueous Medium—Method by Measuring the Oxygen Demand in a Closed Respirometer. International Organization for Standardization: Geneva, Switzerland, 2019],
- ISO 14852 [Determination of the Ultimate Aerobic Biodegradability of Plastic Materials in an Aqueous Medium—Method by Analysis of Evolved Carbon Dioxide. International Organization for Standardization: Geneva, Switzerland, 2021],
- ISO 14853 [Determination of the Ultimate Anaerobic Biodegradability of Plastic Materials in an Aqueous System—Method by Measurement of Biogas Production. International Organization for Standardization: Geneva, Switzerland, 2016], and
- EN 13432 [EN 13432:2000; Packaging—Requirements for packaging recoverable through composting and biodegradation—Test scheme and evaluation criteria for the final acceptance of packaging. European Committee for Standardization: Brussels, Belgium, 2000.]. Despite these advances, implementation remains inconsistent across regions. These gaps highlight that even when test methods exist, enforcement and harmonization lag behind scientific progress. However, third-party auditing and dissemination of guidelines through both policy and academic channels are now the strategy used to ensure compliance and public accountability [439].

In the biomedical domain, the regulatory landscape is equally complex. Standards such as ISO 10993 [Biological Evaluation of Medical Devices—Part 1: Evaluation and Testing within a Risk Management Process. International Organization for Standardization: Geneva, Switzerland, 2018] provide guidance on cytotoxicity, sensitization, irritation, and implantation testing, but these remain largely device-oriented rather than material-oriented, meaning that even “proven” biomaterials must be re-evaluated when introduced in new devices [440]. Agencies including the FDA, EMA, and ASTM are actively developing regulatory frameworks for biomaterials, but the rapid pace of innovation in drug delivery systems, tissue engineering scaffolds, and minimally invasive implants creates challenges for approval pathways [441]. Issues such as long-term toxicity, foreign body responses, protein adsorption, and inflammatory reactions remain difficult to capture fully in preclinical testing, highlighting the need for more rigorous and degradation-aware biocompatibility assessments [442,443]. Establishing harmonized global standards for both biodegradable and biocompatible polymers is therefore essential, not only to accelerate clinical and industrial translation but also to build public trust in these materials.

## 6. Future Prospects

Advances in key areas will shape the future of biodegradable and biocompatible polymers, including the design of smart, multi-responsive materials, integration with bioelectronics and sensors, strategies for clinical translation and industrial scalability, and the adoption of circular economy principles to improve recyclability and sustainability.

### 6.1. Smart and Multi-Responsive Polymers

The next generation of polymer science is being shaped by “smart” polymers: materials engineered to respond dynamically to environmental cues. In agriculture, for example, stimuli-responsive starch-based coatings are being designed for controlled-release fertilizers (CRFs), where nutrient delivery is coupled with soil triggers such as pH, temperature, moisture, ionic strength, and enzymatic activity. By tailoring starch’s biodegradable backbone through chemical modification, coatings can control nutrient release via mechanisms like ionization-driven swelling, enzyme-mediated scission, and thermally induced coil–globule transitions. These innovations promise to synchronize fertilizer release with crop demand, reduce leaching, and improve soil health under realistic field conditions [444]. In biomedical and industrial packaging contexts, similar principles are being applied to develop polymers that can adaptively release therapeutic agents, maintain product integrity, or resist environmental stressors [445,446]. These advances reflect the growing recognition that multi-responsive polymers can couple biodegradability with precise, real-world functionality.

At the same time, future directions for smart polymers must also emphasize biocompatibility to ensure safe and sustained integration with biological systems. Stimuli-sensitive polymers used in medical applications are required to be non-toxic, non-mutagenic, and responsive to diverse physiological cues [447]. Considerable progress has been made in light-, ultrasound-, and redox-responsive polymers, particularly for drug delivery, but challenges remain in tissue penetration, reproducibility, and controlling the diversity of degradation products [448]. Multi-responsive or dual-responsive polymers, which can respond to combinations of external stimuli, are gaining momentum because they provide higher accuracy and controllability. The integration of two or more responsive materials can generate mismatch strain within 3D-printed structures, introducing multidimensional cues for tissue regeneration. Such systems create multifunctional constructs with more favorable mechanical characteristics, and examples include thermo-photo, thermo-magnetic, and thermo-pH polymers that have already shown promise in real physiological environments [449].

Looking ahead, smart biomedical polymer materials are being designed to go beyond passive responsiveness by enabling intelligent functions such as controlled drug release, self-repair, and dynamic tissue support. Therefore, their integration with artificial intelligence and machine learning is expected to optimize responsiveness and improve personalized therapeutic outcomes [450]. Importantly, ongoing research is expanding beyond small molecule drugs toward immunotherapy and regenerative medicine, with polymers being explored as carriers for cells, antibodies, and other biotherapeutics [448]. Taken together, these developments point toward a future in which smart polymers are not only biodegradable and environmentally sustainable but also biocompatible, enabling applications that span agriculture, packaging, and advanced biomedical therapies.

### 6.2. Integration with Bioelectronics and Sensors

A growing area of interest is the integration of biodegradable polymers with bioelectronics, creating transient sensors and devices that function during a therapeutic window before safely degrading [451]. Such systems, including resorbable pressure monitors and smart wound dressings, combine the biocompatibility of polymers with advances in flexible electronics components [452]. Beyond medicine, similar principles could underpin environmental sensors that monitor soil health or pollutant levels and then safely disappear. Trends in this field are advancing quickly, with PLLA (a PLA variant) emerging as a functional material for sensors, implants, or IoT (Internet of Things) devices [453]. Studies across materials science and bioelectronics emphasize design principles for biodegradable, conductive, and elastomeric matrices, including the use of conductive hydrogel interfaces, and component-level strategies (substrates, encapsulants, interconnects) compatible with physiological environments. The key challenges center on preserving conductivity during hydration and degradation, maintaining mechanical compliance with soft tissues, and ensuring that breakdown products are non-toxic [454,455].

With the rise of wearable medical technologies, biomedical polymer materials that are flexible, lightweight, conductive, and biocompatible are expected to find even broader application in sensors, monitoring devices, and smart implants [450]. These systems can enable continuous health tracking and tailored therapeutic interventions, aligning well with the push toward personalized medicine. In wound care, for instance, infection remains one of the most critical challenges, and next-generation dressings are being developed to combine real-time monitoring, early diagnosis, and on-demand therapy. One recent design integrates flexible polydimethylsiloxane-encapsulated electronics with temperature sensors and ultraviolet (UV) light-emitting diodes on the upper layer, while the lower layer consists of a UV-responsive antibacterial hydrogel. This hybrid dressing not only enables continuous monitoring of wound temperature for early infection detection but also triggers localized antibiotic release under in situ UV irradiation, offering a dual therapeutic and diagnostic function [456]. Together, these advances signal that future bioelectronic systems based on biodegradable and biocompatible polymers could reshape both healthcare and environmental monitoring by merging adaptability, sustainability, and patient safety into a single material platform.

### 6.3. Clinical Translation and Industrial Scalability

Despite strong laboratory data, very few biodegradable and biocompatible polymers have progressed to routine clinical use. Future efforts must place greater emphasis on long-term biocompatibility studies, standardized degradation metrics, and scalable, cost-effective manufacturing practices. Regulatory strategies will also need to evolve, as the dynamic and responsive behavior of these advanced materials challenges the current medical device classification systems [457]. Translating the success of biomaterial-based delivery systems from laboratory prototypes to clinically available therapies will require overcoming logistical, economic, and technical barriers. Moving from bench-scale synthesis to industrial production involves not only process optimization but also ensuring consistent quality across large-scale manufacturing. Encouragingly, recent studies on nanoparticle formulations demonstrate that it is possible to maintain reproducibility (showing only about 10% variability in size across production scales that differed by more than 100-fold) when advanced methods such as high-pressure microfluidization are used [458]. In practice, successful scale-up will draw on cross-functional expertise in quality management, textile/process engineering, systems engineering, and regulatory affairs, to bridge experimental prototypes and clinical deployment [459].

At the same time, the manufacturing of biomaterials, particularly for protein and cell-based delivery, presents unique technical hurdles that will shape future directions. Sterilization choices (e.g., filtration or chemical methods) can compromise therapeutic integrity or alter polymer functionality, demanding sterilization-aware design of materials and processes [458]. Furthermore, performance can vary significantly between in vitro and in vivo settings: Resorbable polymers frequently degrade more intricately in vivo, where the tissue environment, surgical handling, and sterilization history can shift erosion mechanisms and kinetics; future designs should purposefully tune mechanics and degradation profiles to stimulate tissue regeneration, while recognizing that “simpler” legacy monomers and polymers still have untapped potential. [459]. Looking forward, ensuring safety and efficacy will demand rigorous preclinical testing, transparent evaluation frameworks, and close collaboration between scientists, industry, and regulatory bodies such as the FDA and EMA [458]. Non-destructive, in vivo-relevant monitoring approaches, such as fluorescence imaging to track polymer erosion (e.g., PLGA) in living or ex vivo models, are proposed to become important tools for reducing the risks of translation [443].

Ultimately, the future of clinical translation and industrial scalability rests on integrating material design with considerations of formulation, in vitro and in vivo testing, regulatory compliance, and industrial processing [460]. By addressing these areas together, biodegradable and biocompatible polymers can move from niche laboratory innovations into reliable, widely accessible clinical and industrial products.

### 6.4. Circular Economy and Recyclability

Finally, the long-term sustainability of biodegradable polymers depends on embedding them within circular economy frameworks. Rather than relying solely on biodegradation, future strategies emphasize closed-loop recycling and upcycling. For example, PLA may be chemically depolymerized back into lactide monomers, and new enzymatic routes show promise for polyhydroxyalkanoates (PHAs) [431]. By developing polymers for regulated end-of-life scenarios, researchers can ensure both performance and environmental sustainability. The future also rests upon sustainable synthesis: polymers created from renewable feedstocks and manufactured utilizing ecologically friendly methods. Recent research highlights breakthroughs in combining lignin, starch, and bacterial fermentation to generate biopolyesters, while employing green solvents and catalysts to reduce energy input [461]. Green chemistry advancements and the use of circular economy ideas are becoming increasingly accepted as vital for the manufacture of low-carbon, sustainable polymer products [450]. These approaches are consistent with global aims for lowering carbon footprints and promoting material circularity.

Science is working to develop materials that can not only biodegrade but also be recycled or reused as part of an evolving circular economy. Such measures assist in closing the loop on waste and resource consumption, matching the larger sustainability goals [5,434]. This viewpoint places biopolymers within broader sustainability strategies, viewing them as enablers of closed-loop systems that reduce both environmental load and economic expense. As a result, the future of biodegradable polymers is linked to changing entire material life cycles rather than just addressing single-application difficulties. Revisions to identification standards and LCA practice are frequently advocated for to enable valid claims, better purchasing signals, and design criteria that reward managed end-of-life rather than nominal biodegradability [431].

## Data Availability

No new data were created or analyzed in this study. Data sharing is not applicable to this article.
